# Estimating large carnivore populations at global scale based on spatial predictions of density and distribution – Application to the jaguar (*Panthera onca*)

**DOI:** 10.1371/journal.pone.0194719

**Published:** 2018-03-26

**Authors:** Włodzimierz Jędrzejewski, Hugh S. Robinson, Maria Abarca, Katherine A. Zeller, Grisel Velasquez, Evi A. D. Paemelaere, Joshua F. Goldberg, Esteban Payan, Rafael Hoogesteijn, Ernesto O. Boede, Krzysztof Schmidt, Margarita Lampo, Ángel L. Viloria, Rafael Carreño, Nathaniel Robinson, Paul M. Lukacs, J. Joshua Nowak, Roberto Salom-Pérez, Franklin Castañeda, Valeria Boron, Howard Quigley

**Affiliations:** 1 Centro de Ecología, Instituto Venezolano de Investigaciones Científicas (IVIC), Caracas, Venezuela; 2 Panthera, New York, United States of America; 3 Wildlife Biology Program, Department of Ecosystem and Conservation Sciences, W. A. Franke College of Forestry and Conservation, University of Montana, Missoula, United States of America; 4 Department of Environmental Conservation, University of Massachusetts, Amherst, United States of America; 5 Evolution, Ecology and Organismal Biology Program, University of California, Riverside, United States of America; 6 Fundación para el Desarrollo de las Ciencias, Físicas, Matemáticas y Naturales–FUDECI, Caracas, Venezuela; 7 Mammal Research Institute, Polish Academy of Sciences, Białowieża, Poland; 8 Department of Forest Management, W.A. Franke College of Forestry and Conservation, University of Montana, Missoula, United States of America; 9 Durrell Institute of Conservation and Ecology, University of Kent, Canterbury, United Kingdom; Oregon State University, UNITED STATES

## Abstract

Broad scale population estimates of declining species are desired for conservation efforts. However, for many secretive species including large carnivores, such estimates are often difficult. Based on published density estimates obtained through camera trapping, presence/absence data, and globally available predictive variables derived from satellite imagery, we modelled density and occurrence of a large carnivore, the jaguar, across the species’ entire range. We then combined these models in a hierarchical framework to estimate the total population. Our models indicate that potential jaguar density is best predicted by measures of primary productivity, with the highest densities in the most productive tropical habitats and a clear declining gradient with distance from the equator. Jaguar distribution, in contrast, is determined by the combined effects of human impacts and environmental factors: probability of jaguar occurrence increased with forest cover, mean temperature, and annual precipitation and declined with increases in human foot print index and human density. Probability of occurrence was also significantly higher for protected areas than outside of them. We estimated the world’s jaguar population at 173,000 (95% CI: 138,000–208,000) individuals, mostly concentrated in the Amazon Basin; elsewhere, populations tend to be small and fragmented. The high number of jaguars results from the large total area still occupied (almost 9 million km^2^) and low human densities (< 1 person/km^2^) coinciding with high primary productivity in the core area of jaguar range. Our results show the importance of protected areas for jaguar persistence. We conclude that combining modelling of density and distribution can reveal ecological patterns and processes at global scales, can provide robust estimates for use in species assessments, and can guide broad-scale conservation actions.

## Introduction

Broad scale population estimates are desired for setting conservation goals and priorities (e.g. for IUCN assessments) [1], [2]. However, for many secretive species that are difficult to census, such estimates are often based on “expert opinion” which is burdened with a high and unquantifiable level of uncertainty [3], [4]. Large carnivores are difficult to census due to their secretive nature but are considered to be declining globally due to human impacts [5]. For the most conspicuous species, i.e. lions *Panthera leo* and cheetah *Acinonyx jubatus*, attempts have been made to apply direct counts and use them for total population estimation [6], [7]. However, for other carnivore species robust population estimates at a global scale do not exist (e.g. for the jaguar *Panthera onca* and the leopard *P*. *pardus* [8], [9]).

When direct censuses are not possible, population estimates may be derived from known species distributions and spatial variation in population densities. However, as densities are usually highly variable in space, a large number of density studies would be needed to reliably estimate population size; similarly precise distributions of species are rarely known. An alternative approach would be to model density variation and distribution and combine spatial predictions of both models to derive population numbers at a given moment. Species distribution models have become a powerful tool in animal conservation. They can help to estimate current species range, identify factors determining species distribution, and indicate ecological corridors [10], [11]. Various distribution models for large carnivores have been proposed recently, most indicating that the probability of carnivore occurrence may depend on environmental conditions but also increasingly on various human impacts [12]–[14]. Unlike distribution, variation in large carnivore density has not been widely analysed due to a scarcity of adequate data. Recently, population density estimates based on camera trap data have become increasingly common for species that are individually identifiable such as jaguars, leopards, and tigers *P*. *tigris* [15–17]. In many early camera-trapping studies, densities were estimated with non-spatial capture-recapture models, which have been criticised recently as leading to overestimation [18], [19]. Spatial capture-recapture models have been shown to produce more accurate density estimates [20] and are slowly replacing non-spatial methods.

In our study we used the profusion of recently published density estimates to gain insight into the factors affecting the density and distribution of the jaguar at a global scale. To increase our sample size for the analysis of spatial variation in population densities, we proposed a method to rescale the estimates obtained with non-spatial methods to the level of densities obtained with spatial models. Using widely available data derived from satellite imagery, we modelled variation in jaguar density across the species’ range. Based on a second, independent presence-absence dataset, we also developed a distribution model and calculated the probability of jaguar occurrence across the Americas. With this approach, we separately revealed mechanisms and factors that determine population densities and distribution of jaguars. Finally, we combined these results in a hierarchical framework to estimate the current total population of jaguar across the entire species’ range.

## Materials and methods

### Study area

**Fig 1:**
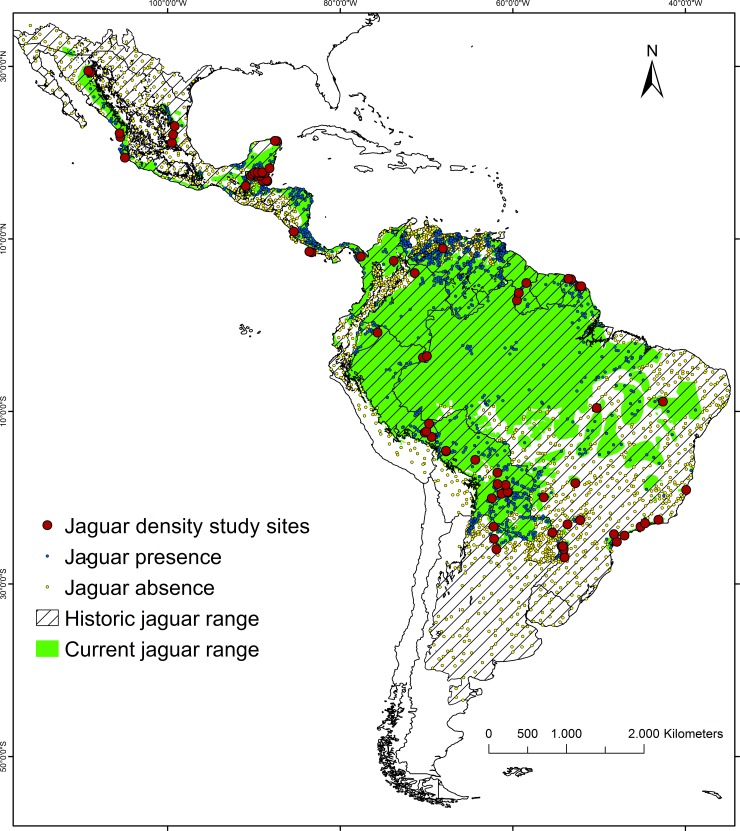
Study area map. Indicated are historical and current jaguar range (see Materials and Methods for definitions and sources for both) and the distribution of density study sites and presence/absence records used for modelling range-wide jaguar density and occurrence.

Our study area covered the entire historical range of the jaguar in South and North America (17.6 million km^2^ [21]) enlarged by a 200 km buffer adjacent to the current jaguar range [8] if outside of the historical range (Fig 1).

### Modelling jaguar density

Jaguar densities were obtained using the published results of 117 camera-trap studies, conducted in 80 different study sites between 2002 and 2014. Our sources included studies published in peer reviewed journals, theses and dissertations, as well as government and non-governmental agency reports (S1 Table). In these studies, jaguar densities were estimated with non-spatial and/or spatially explicit capture-recapture methods. For non-spatial methods, programs CAPTURE [22] or MARK [23] were used. Spatially explicit capture-recapture (SCR) models can be based on maximum likelihood or on a Bayesian framework; the former were usually calculated with programs DENSITY [24–26] or SECR [25], [27] and the latter with programs SPACECAP [28] or SCRbayes [29], [30].

Spatial and non-spatial methods differ in how they calculate densities. To produce a density estimate, non-spatial methods calculate the total number of jaguars and divide that by an estimate of the sampled area, which consists of the study area plus a buffer. This buffer is usually calculated as half the mean maximum distance moved by individuals within the study (hereafter referred as 1/2MMDM) [31]. In SCR methods, density is calculated based on the estimated spatial distribution of animal activity centres, individual detection probabilities, and the spatial distribution of their movements, inferring density directly from these spatial data without requiring an arbitrary buffer [30]. These differences make SCR superior to 1/2MMDM methods [18], [19]. In general, 1/2MMDM methods overestimate densities while SCR methods yield more accurate estimates that are comparable to estimates based on radiotracking [20].

Of the 117 studies analysed here, 59 used non-spatial methods, 53 used both spatial and non-spatial methods simultaneously, and 5 studies presented only spatial methods. We treated 8 studies in which no jaguars were recorded by camera traps as zero density estimates (S1 Table). We assumed that estimates based on 1/2MMDM and SCR methods should be correlated and, if so, both should reflect spatial patterns in the functional relationships between real jaguar densities and environmental conditions at a large geographic scale. To test this assumption, we performed a linear regression between estimates obtained with 1/2MMDM and SCR methods, based on the 53 studies where both methods were used. When SCR estimates were provided based on both frameworks (maximum likelihood and Bayesian), we used an average of the two. We used the output of this regression to predict the SCR estimates for the remaining 59 non-spatial density studies and to rescale them to an SCR level. Some of the original 117 reported densities were repetitions performed in the same study area over a different time period (i.e. multiple years), in these cases we used the mean values for the same location to avoid an overrepresentation of some habitats. In total we used density estimates from 80 locations (Fig 1).

We modelled jaguar density based on 17 candidate spatial variables (S2 Table) in a multiple linear regression. Our candidate variables included 3 anthropogenic and 13 environmental factors, all hypothesized to affect carnivore density. As anthropogenic variables, we used: (a) human population density, which we assume is related to the density of hunters, number of human-carnivore conflicts, and frequency of killing large carnivores and their prey [32–34]; (b) human footprint index, which reflects the degree of anthropogenic habitat changes [35], and (c) classification of protected or non-protected areas. As environmental variables, we used a set of vegetation indices derived from satellite images related to ecosystem productivity and indicating potential abundance of jaguar prey biomass [36], [37]: (a) mean net primary productivity (NPP_MEAN_), (b) mean gross primary productivity (GPP_MEAN_), (c) mean normalized difference vegetation index (NDVI_MEAN_), and (d) mean enhanced vegetation index (EVI_MEAN_). Each of these indices reflects a slightly different component of vegetation productivity with potentially different effects on herbivore populations [36–39]. In addition to mean values, we also used the standard deviations of these variables reflecting environmental variability or seasonality strength, as this may also affect prey availability for jaguars. Additionally, we included mean annual temperature and annual precipitation, which are related to ecosystem productivity and have been shown to have an impact on jaguar distribution [40–42]. Because forests and water are considered important components of jaguar habitat [43], we incorporated mean forest canopy cover and mean and standard deviation of the normalized difference water index (NDWI) [44]. Finally, we included an indicator variable for North and South America (coded as 1 for North and 2 for South America) to account for the smaller jaguar body mass, smaller areas of contiguous habitat, and higher human densities in Central America [45–47]. A detailed description of the predictive anthropogenic and environmental variables used is provided in S2 Table. To test the impact of study design on the precision of density estimate, we also introduced variables characterizing camera-trapping effort of each jaguar study, i.e., number of camera stations, number of study days, total number of trap-nights, and the study area size (S1 Table).

All covariate raster data were standardized to a 1 km × 1 km pixel. We used this resolution to account for the jaguars’ selection for certain habitats, (e.g., those related to water) [48–50]. Using a larger pixel size would dissolve these selectivity patterns, especially in dry areas where jaguars are strictly connected to riparian forests and avoid surrounding dry areas. To each density estimate we assigned a pixel value that corresponded to geographic coordinates of the central part of study area or to the highest concentration of camera traps. Because in some studies detailed maps with exact locations of camera stations and their geographic coordinates were not included we could not calculate averaged pixel values and we assumed that the central pixels were representative of the study area as a whole. We applied a log-transformation to human population density, which had considerable skew [51]. Possible correlations between pairs of variables were evaluated by calculating Pearson correlation coefficients (S3 Table). In the case of correlations above 0.7, the variable less correlated with the dependent variable was removed from the analysis to avoid multicollinearity in the models [52].

We fit all subset models to the density data with the explanatory variables and selected the best model based on Bayesian Information Criterion (BIC) [53–55]. To check if the regression coefficients of the best model were robust, we used bootstrap resampling with 10,000 replications and calculated bias values of the estimates. We tested standard regression assumptions of the best models by examining residual plots (plots of the standardized residuals as a function of standardized predicted values), histograms, and normal probability plots [56]. To estimate the relative importance of each independent variable in the total explained variance of density estimates, we calculated semi-partial correlations with the formula:

$${sr}_{i}^{2}=[1-R^{2}]\times \frac{t_{i}^{2}}{{df}_{res}}$$

where R^2^ is the coefficient of determination, t_i_ is the value of t-statistic for variable *i*, and df_res_ is the number of degrees of freedom for residuals [56].

We projected our top density model across the whole of North—South America.

All spatial analyses were conducted using ArcGIS 10.1 (ESRI Redlands CA, USA). All statistical analyses were performed with SYSTAT 13.0 (Systat Software, Inc., San Jose, CA, USA) and SPSS ver. 20 (IBM SPSS Statistics).

### Predicting jaguar distribution

We gathered jaguar presence/absence data from 4 sources. First, we used 1,266 jaguar records collected by the Wildlife Conservation Society in 1999 and 2006, and published by Zeller [57] as part of a range-delimiting process. However, we corrected these data, classifying them as presence points (993) if they were located inside the most recent, updated in 2014 IUCN jaguar range (Fig 1), [8] and as absence points (273), if they were outside. In the latter case we assumed that jaguars had been extirpated from these areas between 2006 and 2014. The 2014 IUCN jaguar range was updated following the same methodology as used by Zeller [8], [57]. We additionally adjusted it for 15 recently published jaguar records from Mexico, the USA, and Brazil (Fig 1, S1 Data); hereafter this updated and adjusted range is referred to as current jaguar range. Second, we used data from field surveys, including camera trapping, track records, and interviews with hunters and cattle ranchers conducted across Venezuela between 2009 and 2015 (1,238 presence and 540 absence points [14]). Third, we included as presence points the locations of studies of jaguar densities based on camera trapping conducted in South and North America between 2002 and 2014 (72 points with non-zero values, all inside the current jaguar range, Fig 1, S1 Table). Finally, because of the large disparity between the number of presence locations and absence locations, we randomly generated 1000 additional absence points within a buffer of 200 km outside current jaguar range (Fig 1). We reduced densely distributed points, leaving only one if the distance between neighbouring points was less than 5 km to decrease the level of spatial autocorrelation [14], [48]. In total, we used 1,694 presence and 1,683 absence points (Fig 1, S1 Data).

We fit logistic regression models to the presence-absence data and used the same set of candidate predictive variables as for the density model (S2 Table). Again, if highly correlated (r > 0.7), we removed the less predictive variable to avoid multicollinearity (S4 Table). From the set of models fit with all possible combinations of predictive variables, we selected the best model (hereafter: occurrence model) based on Bayesian Information Criterion (BIC). We calculated Nagelkerke's R-Square [58], the area under the receiver operating characteristic (ROC) curve (AUC), and a classification table to evaluate how well the model fit our data. To check if the regression coefficients were robust, we used bootstrap resampling with 10,000 replications and calculated bias values of the estimates. To test the predictive performance of the model, we conducted a 10-fold cross validation with a 75% / 25% data split for our training and evaluation data and we calculated an AUC value for each run [59]. We projected our occurrence model to the entire study area in ArcGIS. The logit values g(*x*) obtained from the best model were converted to probabilities with the function

$$p(x)=\frac{e^{g(x)}}{\left(1+e^{g(x)}\right)}$$

where p(*x*) represents the probability of a 1 km^2^ pixel being occupied [60].

### Estimating jaguar population numbers

We could not derive population numbers directly from our density model because researchers conducting camera trapping studies tend to select less disturbed study areas, where they expect to find jaguars and thus avoid areas with high human impacts. Therefore the predictions of our density model likely represent potential rather than actual jaguar densities. However, human impacts were accounted for in our occurrence model, which was developed with both presence as well as absence points (i.e. where jaguars have been extirpated). To generate a range-wide estimate of the jaguar population, we combined density and occurrence models. The rationale of our method is based on the assumption that variation in potential population density and probabilities of occurrence at large geographic scales are driven by different mechanisms. We assumed that variation in potential density in carnivore populations results from the strong dependence of home range sizes on productivity factors and this relationship is strong even in highly human impacted conditions [61–63]. Probability of occurrence depends on both environmental conditions and human impacts [14] and it reflects the occupancy of potential territories. By multiplying the two models together, a cell with high human impact, and therefore low probability of occurrence, would contribute little to the total population estimate, even in an area with high potential density value. Thus, the product of such multiplication should represent jaguar population density adjusted by human impacts. With these assumptions, we calculated the adjusted jaguar densities by multiplying both models and projected the results over the current jaguar range.

To estimate the total jaguar population size we combined our density and occurrence models in a hierarchical modelling framework using programs R and JAGS, version 4.2.0 (R2Jags) [64]. To make our population model computationally feasible, we resampled our covariate layers (see above) to 10 km x 10 km pixels (see S1 Text for the discussion on how this transformation could influence population size estimates).

Our estimate is hierarchical in that an estimate of density is conditional on the cell first being occupied. We defined the occurrence of jaguars in any given cell (*i*) as a Bernoulli random variable:

$$z_{i}∼Bern(p_{i}),$$

where *p*_i_ is the probability of jaguar occurrence derived from logit g(*x*) values of our top occurrence model, based on the covariates *X*_1-k_: and the corresponding regression coefficients *b*_1-k_

$$logit\;g(x)=b_{0}+b_{1}X_{1}+b_{2}X_{2}+\ldots +b_{k}X_{k}$$

$$p(x)=\frac{e^{g(x)}}{\left(1+e^{g(x)}\right)}$$

Density (*d*) for each cell (*i*) was based on the covariates *Y*_1-k_ of our top density model:

$$\bar{d}=b_{0}+b_{1}Y_{1}+b_{2}Y_{2}+\ldots +b_{k}Y_{k}$$

with assumed normal distribution of *d* around $\bar{d}\;estimate$, with variance σ^2^:

$$d=N(\bar{d},\sigma^{2})$$

Therefore the probable density ($\hat{d}$) for each cell (*i*) in model iteration (*j*) is calculated as:

$$\hat{d_{ij}}=d_{ij}*z_{ij}.$$

Combining our density and occurrence models in this way allowed us to use a distribution of possible beta coefficients for all covariates in both our occurrence and density models, thus incorporating uncertainty for each cell in each iteration of the model. We estimated a posterior distribution of parameters for both models using a Markov Chain Monte Carlo (MCMC) in JAGS (version 4.2.0) through program R (R2Jags) [64]. We iterated the model on a single chain 100,000 times after a burn-in of 1000 and thinned by 100 retaining 990 iterations for each cell. We used normally distributed priors for all covariates with a mean of 0 and precision of 0.001. We standardized all covariate values to have a mean of 0 and a standard deviation of 1.We summed the density estimate for each cell in each iteration to produce a total population estimate (see S2 Text for R code). We calculated posterior standard deviations of our estimate for each cell for each model based on the 990 retained model iteration outputs [65]. We made predictions for current jaguar range, for protected areas within current range, and for the entire historical jaguar range (Fig 1), [8], [21]; in the latter case we assumed that some potentially suitable, although currently not inhabited areas may still exist outside of the current range and potentially they could be recolonized by jaguars in the future.

To validate our method of estimating the global population, we simulated a dataset using similar sampling effort and modelling techniques as in the empirical dataset [65]. We created a data set of 175,000 rows, representing the approximate number of 100 km^2^ cells that makeup historical jaguar range. We then assigned to each row a randomly generated jaguar density and values of six randomly generated continuous and one binomial covariates corresponding to covariates from our density and occurrence models. Finally, to match our sampling level we randomly sampled 0.05% of the cells for density, and 2% of the cells for occurrence. We then used the same MCMC process as above to estimate the simulated population level. We replicated this simulation procedure 100 times. In each replicate, new “population” and covariate values were generated, sampled, and modelled. From the output, we could estimate bias and accuracy of our method [66] (see S2 Text for details).

## Results

### Spatial variation in jaguar density

**Fig 2:**
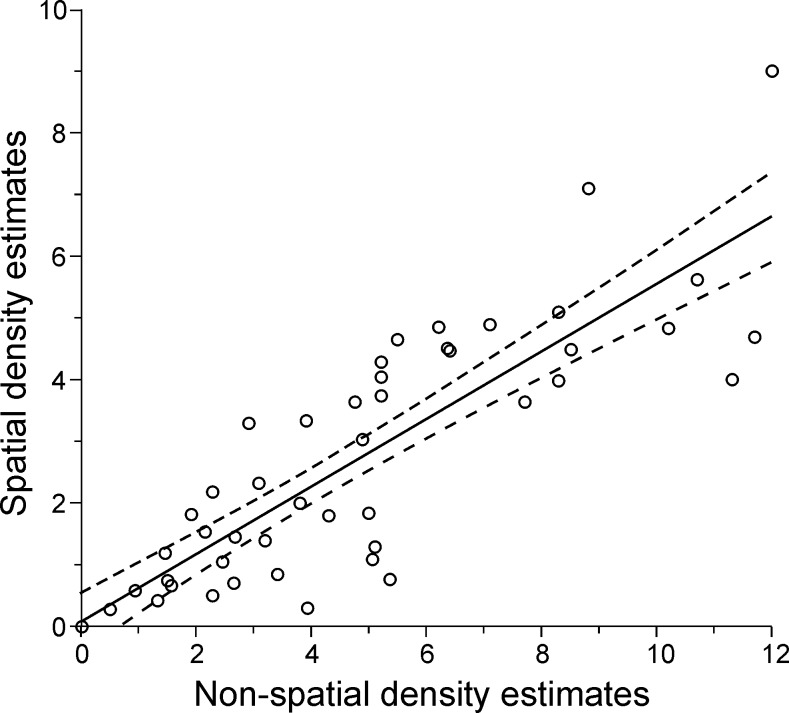
Regression between jaguar density estimates obtained with non-spatial and spatial capture-recapture models. Data points represent 53 published studies in which both non-spatial and spatial density estimates were applied (S1 Table).

Jaguar densities estimated by non-spatial methods (½MMDM) ranged from 0–18.3 per 100 km^2^ and were generally greater than SCR estimates (0–9.0 per 100 km^2^). A regression of SCR and ½MMDM (SCR = 0.07391 + 0.54761 * 1/2MMDM) (Fig 2) was highly significant and predictive (p < 0.001, R^2^ = 0.76, SE = 1.06, N = 53). We used this model to predict SCR density estimates for studies reporting only non-spatial methods, essentially increasing our sample size and standardizing density estimates.

Based on the original (58 studies, 36 study sites) and reconstructed (59 studies, 44 study sites) SCR density estimates we developed a set of regression models explaining spatial variation in jaguar population density. Our top model included four variables: mean annual temperature, mean annual net primary productivity (NPP_MEAN_), standard deviation of annual net primary productivity (NPP_SD_), and a categorical variable distinguishing North and South America (Table 1). This model was highly significant (p < 0.001), explained a relatively high level of variability in density estimates (R^2^ = 0.45, SEE = 1.37, N = 80), and had robust regression coefficients (Table 2). It indicated that productivity factors and mean annual temperature have positive effects on jaguar densities and that at the same environmental conditions jaguar densities are slightly higher in North than in South America. Temperature and NPP_MEAN_ had the greatest ability to explain variability in jaguar density, changing the R^2^ value by 24% and 14% respectively, as indicated by semi-partial correlations sr_i_^2^ (Table 2). Human impact variables were absent from any of the top models (Table 1). In further analyses, we treated predictions of the top model as potential jaguar densities.

**Table 1:** Comparison of multiple linear regression models of jaguar density from 80 sites in North and South America based on values of Bayesian Information Criterion (BIC). Presented are ten best-fitting multiple linear regression models based on 21 spatial variables (three anthropogenic variables, 13 environmental variables, an indicator variable for North and South America (NA-SA), and four variables measuring camera trap effort); definitions of the predictive variables are in S1 and S2 Tables. Density studies were conducted between 2002 and 2014. Bold indicates the model used for spatial prediction of jaguar density.

Model No	Predictive variables	BIC	ΔBIC	BIC weight	R^2^	Significance of covariates
1	**TEMP, NPP**_**MEAN**_**, NPP**_**SD**_**, NA-SA**	298.65	0.00	0.26	0.45	All significant
2	TEMP, NPP_MEAN_, NPP_SD_, NA-SA, N_CamStations	299.78	1.13	0.14	0.48	N_CamStations not significant
3	TEMP, EVI_MEAN_	300.22	1.57	0.12	0.38	All significant
4	TEMP, EVI_MEAN_, N_CamStations	300.61	1.96	0.10	0.41	N_CamStations not significant
5	TEMP, NPP_MEAN_, NPP_SD_, EVI_MEAN_, NA-SA	300.64	1.99	0.09	0.47	EVI_MEAN_ not significant
6	TEMP, EVI_MEAN_, NA-SA	301.22	2.57	0.07	0.40	All significant
7	TEMP, NPP_MEAN_, EVI_MEAN_, NA-SA	301.37	2.72	0.07	0.43	All significant
8	TEMP, NPP_MEAN_, NPP_SD_, EVI_MEAN_, NDVI_SD_, NA-SA	301.51	2.86	0.06	0.49	NDVI_SD_ not significant
9	TEMP, NPP_MEAN_, NPP_SD_, EVI_MEAN_, NA-SA, N_CamStations	301.63	2.98	0.06	0.49	N_CamStations and EVI_MEAN_ not significant
10	TEMP, NPP_MEAN_, NPP_SD_, EVI_MEAN_, NDVI_SD_, NA-SA, N_CamStations	302.94	4.29	0.03	0.51	N_CamStations and NDVI_SD_ not significant

**Table 2:** Parameters of the best-fitting multiple linear regression model of jaguar density from 80 sites in North and South America. Density studies were conducted between 2002 and 2014. Bias and the standard error of the regression coefficients of the bootstrapped model (10,000 replications) are shown; definitions of the predictive variables are in S2 Table.

Effect	Coefficient	Standard Error	t	sr_i_^2^	p-Value	bias	Standard Error*BOO*
CONSTANT	-8.07747	1.92	-4.20		< 0.001	-0.11	1.74
TEMP	0.38911	0.07	5.76	0.24	< 0.001	<0.01	0.05
NPP_MEAN_	0.00136	<0.01	4.40	0.14	< 0.001	<0.01	<0.01
NPP_SD_	0.01026	<0.01	2.68	0.05	0.009	<0.01	<0.01
NA-SA	-1.07356	0.33	-3.27	0.08	0.002	<0.01	0.34

**Fig 3:**
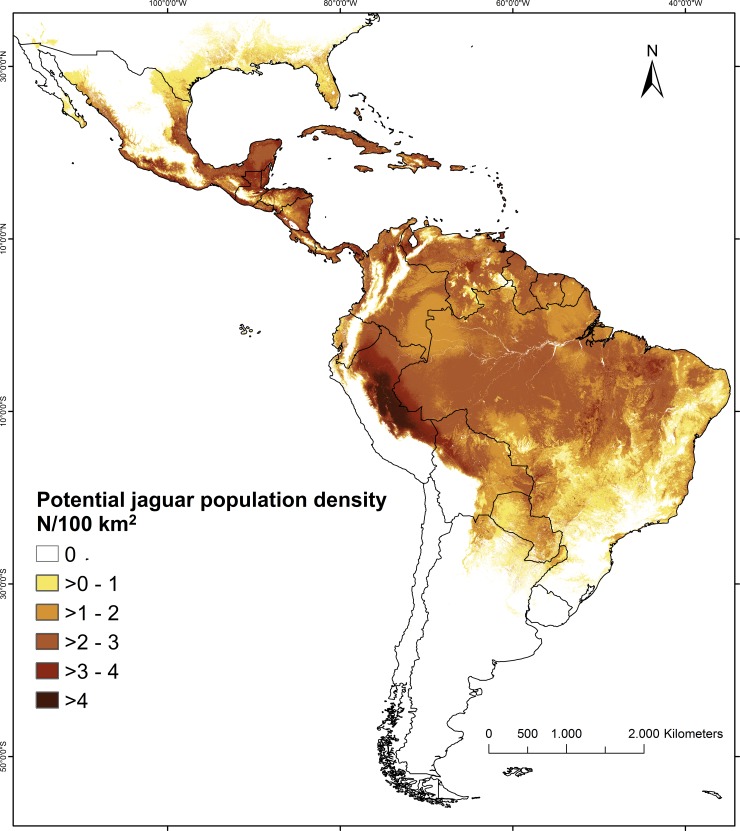
Predicted spatial variation of jaguar potential densities across North and South America. Densities were predicted with our top regression model based on four environmental variables (mean annual temperature, NPP_MEAN_, NPP_SD_, North America–South America code). See also Table 2 for model covariates and associated coefficients.

The spatial prediction of our top model across the Neotropics indicated that jaguars can reach the highest population densities in the most productive, humid areas and the lowest densities in dry areas or in higher altitudes (Fig 3). High potential densities were predicted for most of the Amazon Basin (2–3 jaguars/100 km^2^), and especially for the areas at the base of the Andes in Peru (≥3 jaguars/100 km^2^). High densities were also predicted for the Yucatan Peninsula and eastern coast of Central America (≥3 jaguars/100 km^2^). Our model predicts a clear gradient of declining jaguar potential density with distance from the equator, resulting in low densities at the northernmost and southernmost extremes of historical range. Low or zero densities in high altitude, mountainous regions including the Andes, were predicted (Fig 3). The posterior standard deviation of the potential jaguar densities estimated by our model were low, for most areas lower than 0.6 jaguars/100 km^2^ (S1 Fig).

### Jaguar occurrence

In contrast to the density model, our best supported jaguar occurrence model contained both anthropogenic and environmental variables (Table 3). In our top model, forest cover, temperature, precipitation, and legal protection had positive effects on the probability of jaguar occurrence, while human population density and human footprint index had negative effects (Table 4). With all other variables held constant, the probability of jaguar occurrence was slightly higher in North America than in South America. This model had reasonable predictive performance (p < 0.001, Nagelkerke's R^2^ = 0.624, AUC = 0.912; sensitivity = 0.83, specificity = 0.85, N = 3,377). All coefficients had measurable effect sizes and biases were small (Table 4). In cross validation, the mean AUC value for the estimated probabilities of the smaller subsamples was 0.908 (range 0.889 to 0.922).

**Table 3:** Comparison of the four best-fitting logistic regression models of jaguar presence-absence at 3,155 sites in North and South America, between 2006–2015. Models were fitted with 17 spatial variables (three anthropogenic variables, 13 environmental variables, and North America–South America code); definitions of the predictive variables are in S2 Table. Selection of the best model based on the Bayesian Information Criterion (BIC); additionally Nagelkerke R^2^ and the area under the receiver operating characteristic curve (AUC ROC) are provided. Bold indicates the best model used for spatial prediction of jaguar occurrence.

Model No	Predictive variables	Nagel-kerke R^2^	AUC ROC	BIC	ΔBIC	BIC weight
**1**	**TEMP, PREC, CANOPY, HPDENLG, HFOOTP, PRAR, NA-SA**	**0.624**	**0.912**	2,616.45	0	0.9997
2	TEMP, CANOPY, HPDENLG, HFOOTP, PREC, PRAR	0.619	0.910	2,632.92	16.47	0.0003
3	TEMP, CANOPY, HPDENLG, HFOOTP, NA-SA, PRAR	0.617	0.910	2,639.92	23.47	0.0000
4	TEMP, CANOPY, HPDENLG, NA-SA, PREC, PRAR	0.615	0.908	2,649.89	33.44	0.0000

**Table 4:** Parameters of the best-fitting logistic regression model of jaguar occurrence in North and South America. Definitions of the predictive variables are in S2 Table. Included are biases and *p*-values for regression coefficients of the bootstrapped model.

Parameter	Estimate	Standard Error	Z	p-Value	Odds ratio	Bias	*p*_*BOO*_
CONSTANT	-6.26094	0.47	-13.25	< 0.001		-0.033	< 0.001
TEMP	0.27835	0.02	15.84	< 0.001	1.03	0.001	< 0.001
PREC	0.00046	<0.01	5.45	< 0.001	1.00	<0.001	< 0.001
CANOPY_MEAN_	0.05481	<0.01	18.49	< 0.001	1.06	<0.001	< 0.001
HPDENLG	-0.56917	0.05	-11.20	< 0.001	0.57	0.003	< 0.001
HFOOTP	-0.03480	0.01	-6.32	< 0.001	0.97	<0.001	< 0.001
PRAR	1.19062	0.13	9.06	< 0.001	3.29	0.005	< 0.001
NA-SA	-0.68730	0.14	-4.96	< 0.001	0.50	0.002	< 0.001

**Fig 4:**
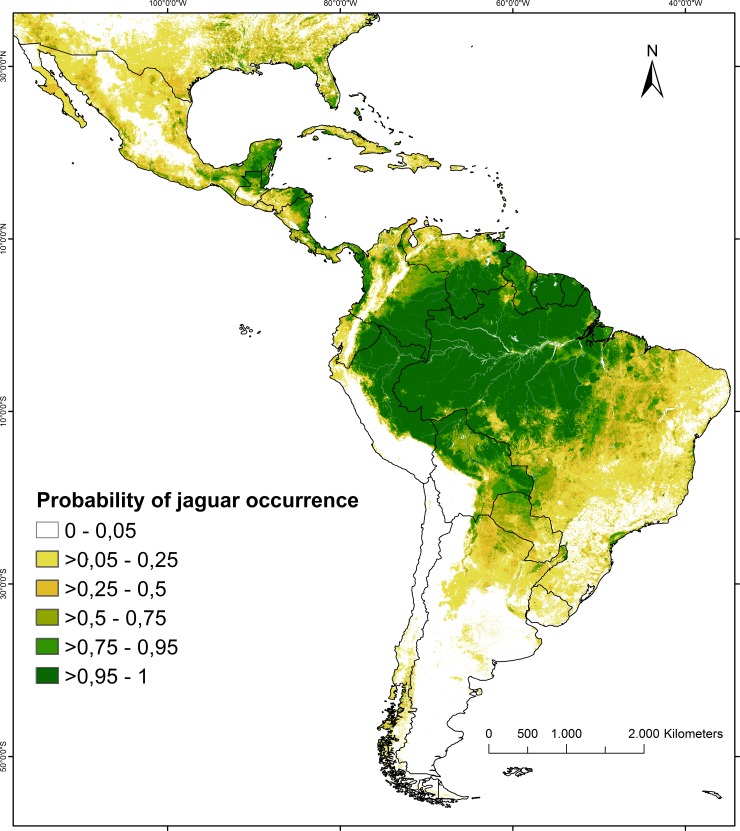
Predicted probability of jaguar occurrence in North and South America. Probability values were predicted by our top occurrence model that included seven spatial variables (mean annual temperature, annual precipitation, forest cover, human density, human footprint index, area protection status, and North America—South America code). See also Table 4 for model covariates and associated coefficients.

Spatial projection of the top occurrence model predicted the highest probabilities of jaguar occurrence in the Amazon basin from the eastern foothills of the Andes to the Atlantic coast and along the eastern coast of Central America (Fig 4). In contrast, the model predicted low probabilities of occurrence in dry areas, in mountain regions at high altitudes, in areas of dense human population (i.e. south-eastern Brazil), and at the northern and southern extremes of historical jaguar range. Our model also suggested a high degree of fragmentation of jaguar range outside the core area of the Amazon Basin (Fig 4). The total area with a probability of jaguar occurrence higher than 0.5 was 9.4 million km^2^, and that with a probability of > 0.9 was 6.2 million km^2^. The posterior standard deviations of the predicted probabilities were generally low. The spatial distribution of these standard deviations indicated that our predictions of jaguar occurrence were least variable for the Amazon basin and somewhat less certain for some parts of Central America (S2 Fig).

### Jaguar population size

In our analysis of simulated data sets, all mean estimates resulting from the hierarchical modelling process were within +/-19.05% of the true value (range 0.01 to 19.05%), with a mean absolute error (MAE) of 6.60%. This indicates that on average the mean population estimate predicted by our model would be within 6.60% of the true simulated population (S2 Text). Our simulations suggest that the hierarchical modelling and the sampling level that were available for us are sufficient to estimate the actual population size with a reasonable degree of error.

**Fig 5:**
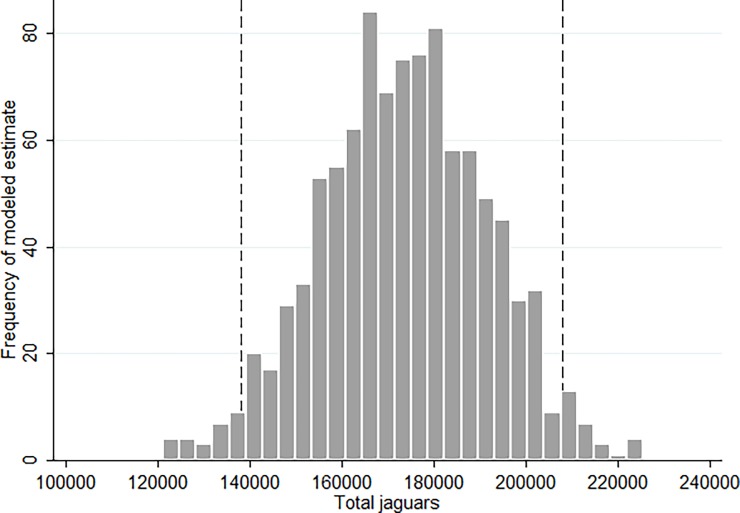
Posterior distribution of range-wide jaguar population estimates. Results obtained from 100,000 iterations of a hierarchical model of jaguar occurrence and density; dashed vertical lines represent a 95% credible interval.

**Fig 6:**
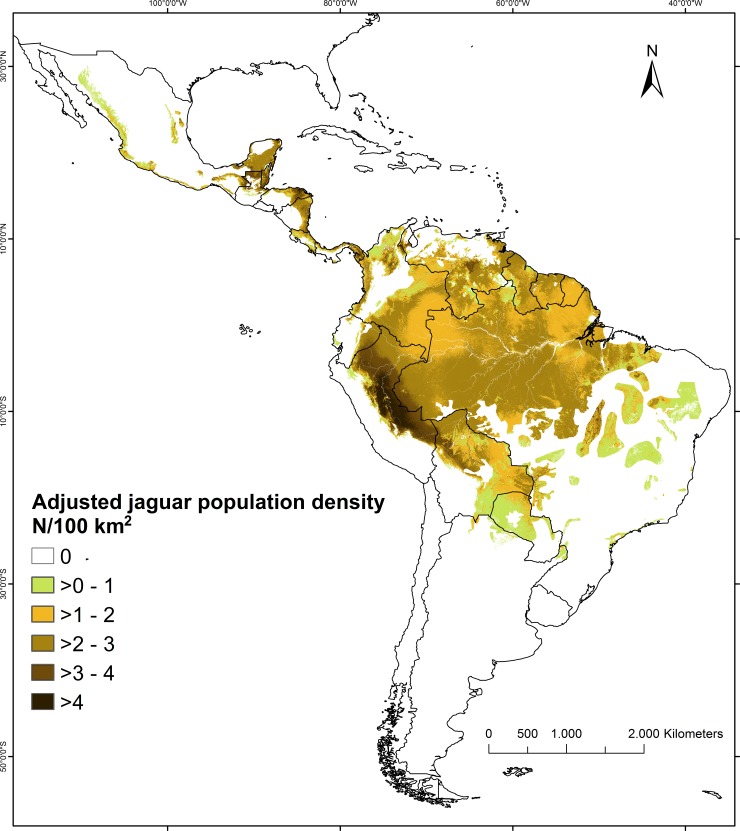
Spatial variation in the mean estimates of adjusted jaguar population densities used for estimating population size within current jaguar range. Adjusted jaguar population densities were estimated using a hierarchical model combining our density and occurrence models and thus accounting for human impacts.

Applying our hierarchical model to our jaguar dataset resulted in a mean posterior estimate of 173,151 jaguars (95% CI: 138,148–208,137) within the current range of the species (8.968 million km^2^), (Fig 5, Table 5). Similarly, we calculated the population size for each country in jaguar range (Table 5). Brazil may possess half of the world’s jaguar population (approx. 86,800), followed by Peru with as many as 22,200. In North America, Mexico is expected to contain the largest population with a mean estimate of approximately 4,300 jaguars. We predicted a population approaching 0 (95% CI 0–4) for the Sonoran region of the United States where single animals were recently observed [67]. The adjusted jaguar population densities, resulting from combining density and occurrence models and restricted to current jaguar range, differed from the potential densities in areas with stronger human impacts (Fig 6). The posterior standard deviations of these predictions were generally low, for the vast majority of jaguar range being lower than 0.6 jaguars/100 km^2^ (S3 Fig), indicating good precision of our estimates.

**Table 5:** Model estimates of occupied area, population size, and mean density of jaguars in the countries of South and North America. Population estimates and 95% credible intervals for each country were derived from hierarchical combination of the best fitting jaguar occurrence and density models based on anthropogenic and environmental variables. Calculations were performed for the area of current jaguar range (Figs 1 and 6).

NR	Country	Current jaguar range area (thousands km^2^)	Mean estimate of jaguar population size (95% Credible Interval)	Mean density N/100 km^2^ (95% CI)
1	Brazil	4,583.6	86,834 (66,865–106,105)	1.89 (1.46–2.31)
2	Peru	739.6	22,210 (17,843–26,788)	3.00 (2.41–3.62)
3	Colombia	872.8	16,598 (11,724–21,311)	1.90 (1.34–2.44)
4	Bolivia	743.1	12,845 (10,260–15,449)	1.73 (1.38–2.08)
5	Venezuela	589.5	11,592 (8,761–14,334)	1.97 (1.49–2.43)
6	Guyana	208.8	4,356 (3,233–5,462)	2.09 (1.55–2.62)
7	Suriname	142.7	3,190 (2,275–4,081)	2.24 (1.59–2.86)
8	Ecuador	93.7	1,969 (1,586–2,359)	2.10 (1.69–2.52)
9	French Guiana	82.2	1,602 (1,097–2,105)	1.95 (1.33–2.56)
10	Paraguay	233.3	1,589 (708–2,497)	0.68 (0.30–1.07)
11	Argentina	76.1	314 (107–550)	0.41 (0.14–0.72)
13	Uruguay	0	0 (0–0)	0.00 (0.00–0.00)
12	Chile	0	0 (0–0)	0.00 (0.00–0.00)
	Total South America	8,365.4	163,098 (127,893–197,494))	1.95 (1.53–2.36)
14	Mexico	339.1	4,343 (3,400–5,383)	1.28 (1.00–1.59)
15	Nicaragua	60.5	1,476 (1,184–1,795)	2.44 (1.96–2.97)
16	Honduras	49.1	1,218 (986–1,447)	2.48 (2.01–2.95)
17	Guatemala	43.1	1,013 (828–1,201)	2.35 (1.92–2.79)
18	Panama	43	869 (692–1,057)	2.02 (1.61–2.46)
19	Costa Rica	38.5	571 (440–716)	1.48 (1.14–1.86)
20	Belize	20.9	563 (463–665)	2.69 (2.22–3.18)
21	United States	7.9	0 (0–4)	0.01 (0.00–0.05)
22	El Salvador	0	0 (0–0)	0.00 (0.00–0.00)
	Total North America	602.1	10,054 (8,352–12,352)	1.67 (1.39–2.05)
	Total South and North Americas	8,967.5	173,151 (138,148–208,137)	1.93 (1.54–2.32)

For comparison, we also estimated the current potential population size across the entire historical range of jaguar (approx. 17,758,200 km^2^), assuming that in the future jaguars may recolonize some potentially suitable areas outside of their current range (compare Figs 1, 4 and 6). Our model estimated 204,650 jaguars (95% CI: 163,742–246,691), suggesting a potential increase of 18% if expansion occurs outside of current range (S5 Table). Finally, we estimated a population of 77,364 jaguars (95% CI: 62,090–92,951) inside protected areas within current range (approx. 3,493,000 km^2^), where presumably jaguars have the highest protection and therefore the greatest chance of persistence (S6 Table).

## Discussion

We have proposed a new method to estimate the range wide population of jaguars, using available density and presence/absence data. The models that we have presented may be used to predict jaguar population densities, probability of occurrence, and population size at a given moment across the Neotropics (i.e. geographic regions, specific protected areas, etc.). Thus, they could be applied to conservation planning of new protected areas or in determining the degree of connectivity between populations. Our results provide a reference for monitoring future trends in jaguar populations.

### Jaguar population numbers

Our estimate of the total jaguar population, approximately 173,000 individuals (CI = 138,000–208,000), was greater than may be expected by many researchers. This estimate may be influenced by the large area (approximately 9 million km^2^) that is still inhabited by jaguars. A large proportion of our estimate was attributed to the forested areas of the Amazon basin, which were characterized by relatively high probabilities of jaguar occurrence and moderate to high densities. In most of this forested area, human population densities are low (< 1 person/km^2^). In such conditions hunting usually has no measurable effect on populations of jaguars and their prey base [33], [34], [68] and jaguars have a high ability to persist, unless deforestation and cattle operations are introduced [14], [69]. Our estimates suggest that jaguars are still likely abundant in some areas, and thus may play an integral role in trophic cascades and prey regulation in neo-tropical ecosystems [70–72]. However, rates of jaguar extirpations continue to increase, mainly due to habitat alteration. During the last 100 years, the range of this species in South America has been reduced to approximately half of its historical distribution [21]. Despite its legal protection in all countries, jaguar populations continue to decline [14]. Our analysis indicated that outside the Amazon basin, jaguar populations are small and highly fragmented and other studies have shown that some of the former strongholds of jaguar, like those in the Atlantic Forest, appear on the verge of extirpation [13]. Therefore, despite our fairly high estimate of the total population, the future of the jaguar is uncertain [8], [73].

Although validating our total population estimate is difficult, the results of our simulations indicated that reasonable estimates at this scale and with this level of sampling are possible (see S2 Text). Our results are also supported by independent studies conducted at smaller scales. Sollmann et al. [74] estimated 52,000 jaguars in the protected areas of Brazil–compared to 46,391 by our study (95% CI: 35,702–56,361; see S6 Table). Based on camera trapping at 16 sites across Mexico, Chávez et al. [75] estimated a total population between 4,000 and 5,000 for the country. Our model predicted 4,343 (95% CI: 3,400–5,383) jaguars for Mexico. Further, Paviolo et al. [13] estimated a jaguar population in the Atlantic Forest (Brazil, Paraguay, Argentina) at 150–300 individuals; our model predicts 336 (95% CI: 136–575) for the same region (approximately 62,400 km^2^). Thus, our results are similar to the prior estimates provided by local studies and our credible intervals contain all those estimates. Alternatively, De la Torre et al. [76] recently estimated the total jaguar population at 64,000 individuals. The marked difference between their estimate and ours may have resulted from the following issues: (a) De la Torre et al. restricted their estimate to 34 subpopulations whereas we modelled the probability of occurrence across all of current range; their subpopulations do not reflect all of the current range (compare Fig 1); (b) De la Torre et al. used 19 density estimates whereas our model was trained using 80 published estimates (see S1 Table); (c) De la Torre et al. used a subjective and simplified selection of density levels applied to different regions and habitats. For instance, one density level was applied to all patches of jaguar range in southern Brazil and Argentina, where varying levels of density have been documented [13]; (d) finally De la Torre et al. applied what we consider a low density (1 jaguar/100 km^2^) to the whole of the “Amazonia” region, which was not based on any field study; this single density was applied to areas from northern Argentina and the Pantanal in the south, through the Amazon Basin to northern Venezuela, where 37 actual studies have been conducted, and where estimated densities range from 0 to 9 jaguars/100 km^2^ (based on spatial methods only, compare S1 Table). In short, De la Torre’s use of a restricted range and low density estimates unsurprisingly results in a lower total estimate than ours.

### Possible biases and limitations of our analyses

Our best density model based on environmental variables explained approximately 45% of the variation in jaguar density estimates throughout their range. The remaining unexplained variation is related to process and sampling variance. In our analysis, density estimates were only slightly influenced by camera trapping study design, such as the size of the study area and the number of camera stations used (Table 1). However, a common factor that increases variation in density estimates is the duration of the monitoring period. Most studies were conducted over a period of < 3 months (to fulfil the assumption of a closed population [19], [31], [77]. Jaguar density estimates from consecutive short periods at the same study area can vary by up to 50% due to seasonal changes in jaguar activity patterns and territory use, which in turn can influence detection probability and abundance estimates [78].

Our density estimates were derived from 80 camera trapping study sites. Although they covered a wide range of habitats and environmental conditions, these estimates may not have captured all of the natural variation in jaguar densities. Further precision in the predictions of models such as ours will be obtained with more replicated and representative density estimates.

Our occurrence model was based on 3,377 presence/absence points, producing robust predictions of jaguar occurrence probability. However, possible bias could result from an uneven distribution of our data, with a higher concentration in some areas of Central America and Venezuela, which potentially could produce spatial autocorrelation and inflation in the estimates of significance of the logistic regression [79]. We partially reduced this possible effect by collapsing all points within 5 km of each other to a single point [14], [48]. The resulting occurrence model validated well, indicating that any bias caused by an uneven distribution of data was rather small. A more likely source of possible bias (overestimation of jaguar range) in our results may come from the fact that some spatial data used as predictive variables may be out-dated (e.g. data on human footprint index from 2004 and data on human densities from 2011, see S2 Table). Updated layers, as well as the addition of other anthropogenic variables to our models, such as road density [80], could possibly increase the precision of the occurrence model predictions. Nevertheless, these problems concern areas with higher human impacts. Because areas with low human impact covered the largest part of jaguar range and had the greatest probability of jaguar occurrence (i.e. the Amazon basin), these errors should not affect our calculations to a great degree.

Our presence/absence data lacked temporally replicated samples at each site, thus we were unable to account for detection probability in our model [81]. However, in the case of the jaguar, non-detection is practically equivalent to true absence, because the jaguar’s large size and its conspicuous behaviour allows its easy detection by hunters, rangers, or cattle producers [14], [82]. Thus, possible bias (underestimation of jaguar range) resulting from this weakness of our data is rather small.

Our models are designed to estimate global distribution and population size at a snapshot in time related to that at which the data was collected. They cannot predict future population changes or population dynamics. New presence/absence data would have to be collected to estimate population increases or declines over time.

### Mechanisms determining variation in population density and distribution

Our analysis reveals the spatial mechanisms that determine jaguar population density and distribution across the species’ range. We demonstrate that jaguar potential (natural) densities are driven by factors related to primary productivity. This finding is in concordance with previous work showing that mean carnivore home range size, which can be viewed as the inverse to density, at large geographic scales is also determined by primary productivity [61–63]. It has been also shown that carnivore densities are driven by productivity and biomass of herbivore prey [83], [84] and that densities of herbivores are determined by primary productivity [85], [86]. Thus, our results indicate a relationship between three trophic levels, providing support for bottom-up regulation of ecosystems, although top-down forces also can act in parallel [87–89]. It has also been shown that primary productivity influences the impacts of carnivores on herbivore populations. In less productive ecosystems carnivores exert a stronger effect over prey populations than in more productive ones [36], [90], [91]. Thus, primary productivity seems to play a crucial role in determining the natural abundance of both herbivores and carnivores as well as the trophic interactions between them. The strong dependence of jaguar potential densities on mean temperature and primary productivity produces a gradient of declining densities with increasing distance from the equator. All these relationships are in agreement with general patterns of biodiversity [92], [93]. Despite the relationship of potential jaguar densities to primary productivity, actual jaguar numbers are often set by human impacts [78], [94]. Our occurrence model accounts for these impacts as the probability of jaguar occurrence declined with increasing human population density. High human densities are associated with increased hunting intensity, human–jaguar conflicts, and negative impacts on prey populations [32–34], [68], [69], [95], [96]. The importance of forest cover and the human footprint index to jaguar occurrence likely reflects the adverse effect of deforestation and other habitat changes on jaguar populations [97]. Conversely, higher temperatures and precipitation, both closely related to primary productivity [40], increase the probability of jaguar occurrence in our distribution model. Greater ecosystem productivity likely increases the capacity of carnivore populations to compensate for human caused mortalities (through enhanced population productivity and density) and thus increases their probability of persistence. Our distribution model predicts that a similar human population density will exert a stronger effect on jaguar populations in dry than in wet habitats. Thus, populations in dry, low productivity areas are more vulnerable to human impacts, as was demonstrated in Venezuela [14].

### Conservation indications and conclusions

Our results show that protected areas have an important positive impact on jaguar persistence. Given the strong, negative association between human activities and the probability of jaguar occurrence, creating more protected areas, like national parks, is among the most important conservation actions for this species and other large carnivores [98], [99].

We conclude that combining modelling of density and distribution can reveal ecological patterns and processes at global scales, can provide robust estimates for use in species assessments (e.g., IUCN), and can guide broad-scale conservation actions, including planning of protected areas and their ecological corridors [100].

## Supporting information

S1 Table Density estimates and other relevant data used for the analysis of spatial variation in jaguar population densities. (DOCX)

S2 Table Candidate predictive variables used in the spatial analysis. (DOCX)

S3 Table Pearson correlation matrix for jaguar density estimates (dependent variable) and a set of predictive variables. (DOCX)

S4 Table Pearson correlation matrix for the jaguar occurrence (dependent variable) and a set of predictive variables. (DOCX)

S5 Table Estimates of jaguar population across historical range. (DOCX)

S6 Table Estimates of jaguar populations within protected areas by current range countries. (DOCX)

S1 Text Discussion of possible effect of increased pixel size of covariate layers on the precision of population size estimates. (DOCX)

S2 Text Simulation to validate hierarchical model; methods and associated R code. (DOC)

S1 Fig Spatial distribution of the posterior standard deviations of jaguar potential density estimates. (DOCX)

S2 Fig Spatial distribution of the posterior standard deviations of estimated probabilities of jaguar occurrence. (DOCX)

S3 Fig Spatial distribution of the posterior standard deviations of adjusted jaguar density estimates. (DOCX)

S1 Data Excel database containing presence—absence data used for modelling probability of jaguar occurrence. (RAR)
