# Supplementary material for: Estimating large carnivore populations at global scale based on spatial predictions of density and distribution – Application to the jaguar (Panthera onca)
Source: PLoS One. 2018 Mar 26;13(3):e0194719. doi: 10.1371/journal.pone.0194719 (PMC5868828; doi:10.1371/journal.pone.0194719)
Supplement: S1 Table — (DOCX) [file pone.0194719.s001.docx]

**Estimating large carnivore populations at global scale based on spatial predictions of density and distribution – application to the jaguar (*Panthera onca*)**

Jędrzejewski W.*, Robinson H.S., Abarca M., Zeller K.A., Velasquez G., Paemelaere E.A.D., Goldberg J.F., Payan E., Hoogesteijn R., Boede E.O., Schmidt K., Lampo M., Viloria Á.L., Carreño R., Robinson N., Lukacs P.M., Nowak J.J., Salom-Pérez R., Castañeda F., Boron V., Quigley H.

*correspondence to: [wjedrzej1@gmail.com](file:///C:\MDoc-Venezuela-S\Papers-manuscripts\Jaguar_Americas_Distr_Dens_Numb_2\PlosBiology\wjedrzej1@gmail.com)

**S1 Table. Density estimates and other relevant data used for the analysis of spatial variation in jaguar population densities.**

For the analysis of spatial variation of density, data were averaged by Study Site ID.

| Report ID | Study Site ID | Year of study | Country | Locality | Lattitude | Longitude | N stations | N study days | Polygon size | Total Trap-Nights | 1/2MMDM density estimate | SCR density estimate (Maximum likelihood) | SCR density estimate (Bayesian methods) | Reference # |
| --- | --- | --- | --- | --- | --- | --- | --- | --- | --- | --- | --- | --- | --- | --- |
| 1 | 1 | 2005 | Argentina | Yabotí | -26.92 | -54.00 | 42 | 45 | 549 | 1,871 | 0.20 |  |  | [1] |
| 2 | 2 | 2009 | Argentina | Copo N.P. | -25.98 | -61.90 | 35 | 52 | 363 | 1,820 | 0.00 | 0.00 | 0.00 | [2] |
| 3 | 3 | 2004 | Argentina | Uruga-í | -25.97 | -54.10 | 34 | 45 | 81 | 1,495 | 0.27 |  |  | [3] |
| 4 | 4 | 2006-2007 | Argentina | PN Iguazú, Iguazú-San Jorge | -25.64 | -54.33 | 49 | 47 | 569 | 2,287 | 1.46 |  | 1.20 | [1,3] |
| 5 | 5 | 2008 | Argentina | Iguazú-Urugua-í, Green Corridor I and II, | -25.63 | -54.21 | 47 | 50 | 1,124 | 2,327 |  |  | 0.89 | [3] |
| 6 | 6 | 2008 | Argentina | Aborigen Region | -24.76 | -62.18 | 30 | 56 | 455 | 1,680 | 0.00 | 0.00 | 0.00 | [2] |
| 7 | 7 | 2010 | Argentina | El Canton | -23.36 | -62.23 | 24 | 50 | 367 | 1,204 | 0.00 | 0.00 | 0.00 | [2] |
| 8 | 8 | 2006 | Belize | Cockscomb Basin | 16.69 | -88.45 | 46 | 62 | 297 | 2,749 | 5.50 |  |  | [4] |
| 9 | 9 | 2002 | Belize | Chiquibul National Park | 16.73 | -88.99 | 18 | 27 | 58 | 486 | 7.48 |  |  | [5] |
| 10 | 10 | 2003 | Belize | Cockscomb basin 2003 | 16.78 | -88.54 | 19 | 65 | 80 |  | 4.82 |  |  | [6] |
| 11 | 10 | 2004 | Belize | Cockscomb basin 2004 | 16.78 | -88.54 | 19 | 72 | 80 |  | 18.29 |  |  | [6] |
| 12 | 10 | 2005 | Belize | Cockscomb basin 2005 | 16.78 | -88.54 | 19 | 77 | 80 |  | 11.45 |  |  | [6] |
| 13 | 11 | 2004 | Belize | Cockscomb Basin | 16.78 | -88.47 | 19 | 62 | 141 | 1,178 | 5.80 |  |  | [4] |
| 14 | 11 | 2005 | Belize | Cockscomb Basin | 16.78 | -88.47 | 19 | 62 | 141 | 1,178 | 5.80 |  |  | [4] |
| 15 | 11 | 2006 | Belize | Cockscomb Basin | 16.78 | -88.47 | 19 | 62 | 141 | 1,178 | 7.00 |  |  | [4] |
| 16 | 12 | 2002 | Belize | Cockscomb Basin | 16.81 | -88.55 | 20 | 59 | 84 | 1,180 | 8.80 |  |  | [5, 6] |
| 17 | 13 | 2008-2009 | Belize | Hill Bank | 17.61 | -88.79 | 20 | 81 | 129 | 890 | 6.20 | 6.00 | 3.70 | [7] |
| 18 | 13 | 2010 | Belize | Hill Bank | 17.61 | -88.79 | 20 | 98 | 129 | 1,069 | 3.90 | 3.80 | 2.90 | [7] |
| 19 | 13 | 2011 | Belize | Hill Bank | 17.61 | -88.79 | 20 | 81 | 129 | 1,541 | 5.50 | 5.30 | 4.00 | [7] |
| 20 | 13 | 2012 | Belize | Hill Bank | 17.61 | -88.79 | 20 | 91 | 129 | 1,419 | 2.90 | 4.00 | 2.60 | [7] |
| 21 | 13 | 2013 | Belize | Hill Bank | 17.61 | -88.79 | 26 | 69 | 129 | 1,606 | 3.80 | 2.10 | 1.90 | [7] |
| 22 | 14 | 2013 | Belize | Gallon Jug | 17.64 | -89.11 | 28 | 84 | 175 | 2,017 | 5.00 | 1.80 | 1.90 | [7] |
| 23 | 15 | 2004 | Belize | Gallon Jug Estate | 17.64 | -89.06 | 28 | 62 | 105 |  | 11.28 |  |  | [8-10] |
| 24 | 15 | 2005 | Belize | Gallon Jug Estate | 17.64 | -89.06 | 24 | 62 | 95 |  | 8.82 |  |  | [8-10] |
| 25 | 16 | 2008-2009 | Belize | La Milpa | 17.73 | -89.05 | 40 | 90 | 146 | 2,579 | 5.20 | 4.50 | 3.00 | [7] |
| 26 | 16 | 2010 | Belize | La Milpa | 17.73 | -89.05 | 20 | 87 | 146 | 776 | 3.40 | 0.90 | 0.80 | [7] |
| 27 | 16 | 2011 | Belize | La Milpa | 17.73 | -89.05 | 20 | 63 | 146 | 1,173 | 5.20 | 3.80 | 4.30 | [7] |
| 28 | 16 | 2012 | Belize | La Milpa | 17.73 | -89.05 | 20 | 71 | 146 | 1,347 | 4.30 | 1.70 | 1.90 | [7] |
| 29 | 16 | 2013 | Belize | La Milpa | 17.73 | -89.05 | 22 | 79 | 146 | 1,665 | 1.50 | 0.60 | 0.90 | [7] |
| 30 | 17 | 2005 | Belize | Fireburn | 18.19 | -88.20 | 16 | 63 | 55 |  | 5.30 |  |  | [9,11] |
| 31 | 18 | 2004 | Bolivia | Guanaco II, Kaa-Iya II, Bolivia | -20.07 | -62.44 | 18 | 60 | 62 | 1,080 | 2.14 |  | 1.54 | [12,13] |
| 32 | 18 | 2003-2004 | Bolivia | Guanaco I, Kaa-Iya I, Bolivia | -20.07 | -62.44 | 16 | 60 | 49 | 960 | 3.21 |  |  | [13-15] |
| 33 | 19 | 2002-2003 | Bolivia | Cerro cortado II, Gran Chaco National Park | -19.54 | -61.32 | 28 | 60 | 49 | 1,680 | 5.37 | 0.90 | 0.63 | [13,16] |
| 34 | 19 | 2002 | Bolivia | Cerro cortado I, Gran Chaco Nationa Park | -19.54 | -61.32 | 38 | 60 | 52 | 2,280 | 5.11 | 1.46 | 1.14 | [13,17] |
| 35 | 20 | 2006-2007 | Bolivia | Palmar-Ravelo II, Kaa-Iya | -19.42 | -60.53 |  |  | 434 | 3,330 | 0.94 | 0.58 | 0.61 | [13,18,19] |
| 36 | 20 | 2006 | Bolivia | Palmar I, Kaa-Iya 2006 | -19.42 | -60.53 | 23 | 61 | 71 | 1,365 | 1.32 |  | 0.42 | [13,18,20,21] |
| 37 | 21 | 2003 | Bolivia | Ravelo I, Gran Chaco Nationa Park | -19.32 | -60.61 | 36 | 60 | 102 | 2,160 | 2.27 | 0.46 | 0.56 | [13,22] |
| 38 | 21 | 2003 | Bolivia | Ravelo II, Gran Chaco Nationa Park | -19.32 | -60.61 | 36 | 60 | 101 | 1,320 | 1.57 | 0.72 | 0.63 | [13,23,24] |
| 39 | 22 | 2002 | Bolivia | Tucavaca I, Gran Chaco Nationa Park | -18.52 | -60.82 | 32 | 60 | 130 | 1,920 | 2.46 | 0.99 | 1.11 | [13,25] |
| 40 | 22 | 2003 | Bolivia | Tucavaca II, Gran Chaco Nationa Park | -18.52 | -60.82 | 16 | 60 | 49 | 1,560 | 3.10 |  |  | [13,26] |
| 41 | 22 | 2004 | Bolivia | Tucavaca III, Gran Chaco Nationa Park | -18.52 | -60.82 | 16 | 60 | 49 | 1,560 | 3.20 |  | 1.39 | [13,27] |
| 42 | 23 | 2005 | Bolivia | Estacion Isoso I, Kaa-Iya 2005 | -18.42 | -61.77 | 22 | 56 | 48 | 1,232 | 1.90 |  | 1.82 | [13,28] |
| 43 | 23 | 2006 | Bolivia | Estacion Isoso II, Kaa-Iya 2006 | -18.42 | -61.77 | 20 | 64 | 51 | 1,268 | 3.93 |  | 0.31 | [13,21,29] |
| 44 | 24 |  | Bolivia | San Miguelito | -17.10 | -61.78 | 25 | 60 | 54 |  | 4.23 |  |  | [8,9,30] |
| 45 | 25 | 2006 | Bolivia | EL Encanto, Santa Cruz | -15.60 | -64.36 | 20 | 60 | 36 |  | 5.66 |  |  | [8,9,31] |
| 46 | 26 | 2002 | Bolivia | Madidi NP | -14.63 | -67.73 | 66 | 28 | 228 | 1,848 | 2.84 |  |  | [5] |
| 47 | 27 | 2002 | Bolivia | Ríos Tuichi and Hondo, Madidi NP | -14.55 | -67.75 | 32 | 29 | 77 | 896 | 1.68 |  |  | [32] |
| 48 | 28 | 2010-2011 | Brazil | Ilha do Cardoso | -25.11 | -47.94 | 8 | 93 | 55 | 744 | 0.00 | 0.00 | 0.00 | [3] |
| 49 | 29 | 2012 | Brazil | Juréia-Itatins | -24.38 | -47.06 | 21 | 118 | 258 | 2,483 | 0.00 | 0.00 | 0.00 | [3] |
| 50 | 30 | 2011 | Brazil | Intervales-Petar | -24.22 | -48.29 | 24 | 113 | 292 | 2,712 |  |  | 0.66 | [3] |
| 51 | 31 | 2013 | Brazil | Santa Virginia | -23.36 | -45.20 | 26 | 97 | 32 | 2,512 | 0.00 | 0.00 | 0.00 | [3] |
| 52 | 32 | 2008-2009 | Brazil | Ivinhema | -23.08 | -53.66 | 13 | 115 | 330 | 1,495 |  |  | 1.66 | [3] |
| 53 | 33 | 2008-2009 | Brazil | Serra da Bocaina | -22.96 | -44.67 | 26 | 117 |  | 3,054 | 0.00 | 0.00 | 0.00 | [3] |
| 54 | 34 | 2003 | Brazil | Moro do Diablo | -22.61 | -52.20 | 36 | 40 | 225 | 1,440 |  |  | 2.39 | [3,33] |
| 55 | 35 | 2010-2011 | Brazil | Serra dos Órgãos I, II, III and IV. | -22.56 | -43.13 | 44 | 104 | 390 | 4,597 | 0.00 | 0.00 | 0.00 | [3] |
| 56 | 36 | 2003 | Brazil | Pantanal, Fazenda Sete 2004 | -19.95 | -56.42 | 16 | 60 | 110 | 960 | 11.70 |  |  | [34] |
| 57 | 36 | 2004 | Brazil | Pantanal, Fazenda Sete 2003 | -19.95 | -56.42 | 42 | 20 | 165 | 960 | 10.30 |  |  | [34] |
| 58 | 37 | 2005-2006 | Brazil | Vale NR I, II, III, IV and V | -19.09 | -39.91 | 30 | 30 |  | 3,032 |  |  | 2.42 | [3] |
| 59 | 38 | 2002 | Brazil | Emas N.P. Cerrado, Brazil | -18.32 | -52.75 | 29 | 62 | 500 |  | 2.00 |  |  | [35] |
| 60 | 38 | 2008 | Brazil | Emas National Park | -18.32 | -52.75 | 119 | 85 | 1320 |  | 0.51 |  | 0.29 | [36] |
| 61 | 39 |  | Brazil | Santa Fé Ranch | -9.60 | -50.28 |  |  | 80 |  | 2.59 |  |  | [8] |
| 62 | 39 | 2007 | Brazil | Santa Fé Ranch | -9.60 | -50.28 | 21 | 80 | 53 | 1,680 | 3.99 |  |  | [37] |
| 63 | 40 | 2007 | Brazil | Caatinga-Serra da Capivara | -8.86 | -42.61 | 20 | 84 | 205 | 1,450 | 2.67 |  | 1.45 | [38,39] |
| 64 | 41 | 2006 | Colombia | Amacayacu | -3.75 | -70.42 | 18 | 100 | 32 | 1,793 | 4.20 |  |  | [40] |
| 65 | 42 | 2007 | Colombia | Calderon river valley | -3.59 | -69.96 | 27 | 42 | 75,5 | 1,132 | 2.70 |  |  | [40] |
| 66 | 43 | 2014 | Colombia | Orinoco river | 6.05 | -71.37 | 52 | 70 | 151 | 2,457 | 2.27 | 2.19 |  | [41] |
| 67 | 44 | 2014 | Colombia | Magdalena river | 7.47 | -73.80 | 47 | 75 | 155 | 2,251 | 4.88 | 3.04 |  | [41] |
| 68 | 45 | 2007 | Costa Rica | Carbonera y la Leona. Límites con PN Corcovado | 8.45 | -83.33 | 134 | 35 | 102 | 4,690 | 2.00 |  |  | [42] |
| 69 | 46 | 2002-2003 | Costa Rica | Corcovado | 8.53 | -83.58 | 11 | 30 | 30 | 363 | 6.98 |  |  | [43] |
| 70 | 47 | 2005-2006 | Costa Rica | Sector San Cristóbal, dentro del área de conservación Guanacaste, Costa Rica | 10.86 | -85.37 | 15 | 43 |  | 645 | 5.76 |  |  | [44] |
| 71 | 48 |  | Ecuador | Yasuní ITT | -0.85 | -75.67 | 32 | 64 | 58 |  | 2.20 |  |  | [9,45] |
| 72 | 49 | 2005 | Guatemala | Tikal | 17.23 | -89.62 | 15 | 34 | 39 | 510 | 6.63 |  |  | [9,46] |
| 73 | 50 | 2009 | Guatemala | Laguna del Tigre, Sur Corredor Biológico, Portion of Consecion AFISAP | 17.30 | -90.35 | 24 | 49 | 72 | 1,127 | 6.36 | 4.52 |  | [9,47**]** |
| 74 | 51 | 2009 | Guatemala | Melchor de Mencos | 17.34 | -89.27 | 23 | 45 | 67 | 1,035 | 6.04 |  |  | [9,48] |
| 75 | 52 | 2008 | Guatemala | Carmelita-AFISAP | 17.50 | -90.17 | 20 | 45 | 51 | 900 | 11.28 |  |  | [9,49] |
| 76 | 53 | 2007 | Guatemala | La Gloria-Lechugal | 17.58 | -89.78 | 33 | 46 | 128 | 1,455 | 1.54 |  |  | [9,50] |
| 77 | 54 |  | Guatemala | Mirador, Oeste | 17.71 | -89.92 | 33 | 47 | 94 | 1,551 | 1.99 |  |  | [51] |
| 78 | 55 | 2008 | Guatemala | Dos Lagunas Río Azul, Mirador Río Azul N.P. | 17.71 | -89.54 | 25 | 47 | 39 | 1,175 | 11.14 |  |  | [9,52] |
| 79 | 56 | 2010 | Guayana Francesa | Reserve des Nouragues | 4.17 | -52.67 | 20 |  |  | 1,870 | 4.40 |  |  | [53-55] |
| 80 | 57 | 2009 | Guayana Francesa | Montagne de Kaw | 4.58 | -52.33 | 20 |  |  | 1,530 | 2.90 |  |  | [53-55] |
| 81 | 58 | 2008 | Guayana Francesa | Counami Forest, French Guyana | 5.30 | -53.09 | 19 | 90 | 60 | 1,690 | 3.30 |  |  | [53-55] |
| 82 | 59 | 2007 | Guayana Francesa | Montagne de Fer, French Guyana | 5.33 | -53.53 | 19 | 90 | 70 | 1,656 | 5.10 |  |  | [53-55] |
| 83 | 60 | 2012 | Guyana | Dadanawa | 2.87 | -59.52 | 48 | 30 | 112 | 1,466 | 1.60 |  |  | [56] |
| 84 | 61 | 2011 | Guyana | Karanambu | 3.75 | -59.31 | 60 | 30 | 80 | 1,972 | 2.70 |  |  | [56] |
| 85 | 62 | 2012-2013 | Guyana | Charabaru Conssesion | 4.89 | -58.44 | 34 | 46 | 77 | 1,593 | 6.40 | 4.48 |  | [57] |
| 86 | 63 | 2007 | Mexico | Montes Azules 2007 wet | 16.14 | -90.96 | 32 | 60 | 81 | 935 | 2.60 |  |  | [58] |
| 87 | 63 | 2007 | Mexico | Montes Azules 2007 wet | 16.14 | -90.96 | 33 | 60 | 82 | 1,920 | 4.60 |  |  | [58] |
| 88 | 63 | 2008 | Mexico | Montes Azules 2007 wet | 16.14 | -90.96 | 42 | 60 | 77 | 2,520 | 2.60 |  |  | [58] |
| 89 | 64 | 2008 | Mexico | Chamela-Cuixamala Jalisco | 19.42 | -104.95 | 29 | 50 | 72 | 725 | 5.38 |  |  | [59] |
| 90 | 65 | 2006-2007 | Mexico | Queretaro | 21.16 | -99.51 | 29.54  ** | 120 | 147 |  | 0.75 |  |  | [60] |
| 91 | 66 | 2008 | Mexico | Reserva Ecológica El Eden | 21.35 | -87.35 | 27 | 65 | 63 | 1,755 | 2.65 |  | 0.70 | [61] |
| 92 | 66 | 2010 | Mexico | Reserva Ecológica El Eden | 21.35 | -87.35 | 24 | 48 | 48 | 1,152 | 5.05 |  | 1.10 | [61] |
| 93 | 66 | 2011 | Mexico | Reserva Ecológica El Eden | 21.35 | -87.35 | 22 | 82 | 54 | 1,804 | 4.76 |  | 3.65 | [61] |
| 94 | 66 | 2012 | Mexico | Reserva Ecológica El Eden | 21.35 | -87.35 | 24 | 72 | 75 | 1,728 | 3.08 |  | 2.33 | [61] |
| 95 | 67 | 2004 | Mexico | Reserva de Biosfera Río Lagartos, Yucatan | 21.37 | -87.58 | 24 | 34 | 86 | 816 | 3.28 |  |  | [62] |
| 96 | 67 | 2005 | Mexico | Reserva de Biosfera Río Lagartos, Yucatan | 21.37 | -87.58 | 13 | 89 | 74 | 1,157 | 1.82 |  |  | [62] |
| 97 | 68 | 2011 | Mexico | Marismas Nacionales Nayarit (sector sur) | 21.82 | -105.48 | 18 | 43 | 59 | 774 | 5.90 |  |  | [63] |
| 98 | 69 | 2008 | Mexico | San Luis Potosí 2008 | 22.08 | -99.38 | 27 | 31 | 53 | 837 | 3.20 |  |  | [64] |
| 99 | 70 | 2011 | Mexico | Marismas Nacionales Nayarit (Sector norte) | 22.25 | -105.57 | 17 | 43 | 76 | 731 | 2.50 |  |  | [63] |
| 100 | 71 | 2013-2014 | Mexico | Tamaulipas, Gómez Farías | 23.09 | -99.18 | 22 | 360 | 21,6 | 7,920 | 5.90 |  |  | [65] |
| 101 | 72 | 2005 | Mexico | Sonora | 29.30 | -108.94 | 26 | 60 | 100 | 1,560 | 0.56 |  |  | [66] |
| 102 | 73 | 2009-2010 | Mexico | Sonora | 29.49 | -109.20 | 58 | 480 | 330 | 7,718 | 1.05 |  |  | [67] |
| 103 | 74 | 2005 | Panama | Darién | 7.96 | -77.60 | 23 | 35 | 67 | 805 | 1.63 |  |  | [68] |
| 104 | 74 | 2006 | Panama | Darién | 7.96 | -77.60 | 22 | 50 | 110 | 1,100 | 5.55 |  |  | [68] |
| 105 | 75 | 2011 | Paraguay | Mbaracayú | -24.03 | -55.41 | 25 | 74 | 188 | 1,844 | 1.29 |  |  | [3] |
| 106 | 76 | 2007 | Peru | Malinowsky/Bahuaja Sonene N.P., Tambopata | -12.95 | -69.42 | 43 | 62 | 52 | 2,585 | 8.80 |  | 7.10 | [69] |
| 107 | 77 | 2010 | Peru | CM2 | -12.45 | -70.20 | 30 | 104 | 196 | 3,131 | 5.20 |  | 4.30 | [69] |
| 108 | 78 | 2005 | Peru | Los Amigos 2005 | -12.32 | -70.03 | 24 | 62 | 56 | 1,478 | 12.00 |  | 9.00 | [69] |
| 109 | 78 | 2006 | Peru | Los Amigos 2006 | -12.32 | -70.03 | 40 | 62 | 56 | 2,059 | 8.50 |  | 4.50 | [69] |
| 110 | 78 | 2007 | Peru | Los Amigos 2007 | -12.32 | -70.03 | 40 | 62 | 56 | 2,510 | 11.30 |  | 4.00 | [69] |
| 111 | 79 | 2009-2010 | Peru | Espinoza | -11.42 | -69.70 | 38 | 122 | 250 | 3,460 | 7.10 |  | 4.90 | [69] |
| 112 | 80 | 2013 | Venezuela | Hato Piñero | 8.90 | -68.11 | 48 | 47 | 143 | 2,256 | 7.70 | 3.65 |  | [70] |
| 113 | 80 | 2013 | Venezuela | Hato Piñero | 8.90 | -68.11 | 56 | 40 | 135 | 2,240 | 10.20 | 4.84 |  | [70] |
| 114 | 80 | 2013 | Venezuela | Hato Piñero | 8.90 | -68.11 | 27 | 65 | 114 | 1,755 | 10.70 | 5.62 |  | [70] |
| 115 | 80 | 2014 | Venezuela | Hato Piñero | 8.90 | -68.11 | 42 | 69 | 157 | 2,898 | 8.30 | 3.99 |  | [70] |
| 116 | 80 | 2014 | Venezuela | Hato Piñero | 8.90 | -68.11 | 32 | 57 | 146 | 1,824 | 8.30 | 5.11 |  | [70] |
| 117 | 80 | 2014 | Venezuela | Hato Piñero | 8.90 | -68.11 | 31 | 99 | 114 | 3,069 | 11.70 | 4.69 |  | [70] |

** In the spatial analysis it was the only missing value for this variable, thus we substituted this cell by the mean number of camera stations from all other studies

**References for S1 Table:**

1. Paviolo A, De Angelo CD, Di Blanco YE, Di Bitetti MS. Jaguar *Panthera onca* population decline in the Upper Paraná Atlantic Forest of Argentina and Brazil. Oryx. 2008; 42: 554-561. doi:10.1017/S003060530800064185
2. Quiroga VA, Boaglio GI, Noss AJ, Di Bitetti MS. Critical population status of the jaguar *Panthera onca* in the Argentine Chaco: camera-trap surveys suggest recent collapse and imminent regional extinction. Oryx. 2014; 48: 141-148.
3. Paviolo A, De Angelo C, Ferraz KM, Morato RG, Pardo JM, Srbek-Araujo AC, de Mello Beisiegel B, Lima F, Sana D, da Silva MX. A biodiversity hotspot losing its top predator: The challenge of jaguar conservation in the Atlantic Forest of South America. Sci Rep. 2016; 6: 37147. doi:10.1038/srep37147
4. Foster R. The ecology of jaguar (*Panthera onca*) in a human-influenced landscape: University of Southhampton; 2008.
5. Silver SC, Ostro LET, Marsh LK, Maffei L, Noss AJ, Kelly MJ, Wallace RB, Gomez H, G. Ayala. The use of camera traps for estimating jaguar *Panthera onca* abundance and density using capture/recapture analysis. Oryx. 2004; 38: 148-154. doi:10.1017/s0030605304000286
6. Harmsen BJ. The use of camera traps for estimating abundance and studying the ecology of jaguars (*Panthera onca*). Southampton, UK: University of Southampton; 2006.
7. Kelly MJ, Rowe C. Analysis of 5-years of data from Rio Bravo Conservation and Management Area (RBCMA) and one year of data from Gallon Jug/Yalbac Ranch, on trap rates and occupancy for predators and prey, including jaguar density estimates in unlogged versus sustainably logged areas. Progress Report for: Rio Bravo Conservation and Management Area, Programme for Belize. 2014.
8. Maffei L, Noss AJ, Silver SC, Kelly MJ. Abundance/density case study: Jaguars in the Americas. In: O’Connell AF, Nichols JD, Karanth K, editors. Camera traps in animal ecology. Japan: Springer; 2011. pp. 119-144.
9. Tobler MW, Powell GVN. Estimating jaguar densities with camera traps: Problems with current designs and recommendations for future studies. Biol Conserv. 2013; 159: 109-118. doi:10.1016/j.biocon.2012.12.009.
10. Miller CM. Jaguar density in Gallon Jug Estate, Belize. Wildlife Conservation Society, 2005.
11. Miller CM. Jaguar Density in Fireburn, Belize. Wildlife Conservation Society and belize Forest Department, 2006.
12. Cuéllar E, Segundo J, Castro G, Noss A. Jaguar and other mammal camera trap survey Guanaco II, Guanaco field camp (20°3'03″S, 62°26'04″W), Kaa-Iya del Gran Chaco National Park, 18 August-18 October 2004. Wildlife Conservation Society. 2004.
13. Noss AJ, Gardner B, Maffei L, Cuéllar E, Montaño R, Romero-Muñoz A, Sollman R, O'Connell AF. Comparison of density estimation methods for mammal populations with camera traps in the Kaa-Iya del Gran Chaco landscape. Anim Conserv. 2012; 15: 527-535. doi:10.1111/j.1469-1795.2012.00545.x
14. Cuéllar E, Segundo J, Castro G, Barrientos J, Healy J, Hesse A, Noss A. Jaguar and other mammal camera trap survey Guanaco I, Guanaco field camp (20° 03' 03" S, 62° 26' 04" W). Kaa-Iya del Gran Chaco National Park 19 December 2003-16 February 2004. Santa Cruz, Bolivia, Wildlife Conservation Society. 2004; Technical Report #104.
15. Cuéllar E. Primer muestreo de jaguares *Panthera onca* en Pampa, zona Guanacos, Parque Nacional Kaa-Iya del Gran Chaco. MEMORIAS: Manejo de Fauna silvestre en Amazonia y Latinoamérica. 2004; 4:158-165
16. Maffei L, Barrientos J, Mendoza F, Ity E, Noss A. Jaguar and other mammal camera trap survey Cerro II, Cerro Cortado field camp (19° 31' 36 ″S, 61°18' 36 ″W). Kaa-Iya del Gran Chaco National Park: Wildlife Conservation Society, 2003 28 November 2002-28 January 2003.
17. Maffei L, Barrientos J, Mendoza F, Ity E, Noss A. Jaguar and other mammal camera trap survey Cerro I, Cerro Cortado field camp (19° 31'36'' S, 61°18' 36'' W), 1 April–30 May 2002. Santa Cruz: Capitanía de Alto y Bajo Izozog and Wildlife Conservation Society. Kaa-Iya del Gran Chaco National Park 2002; Technical Paper No. 84.
18. Romero-Muñoz A, Noss AJ, Maffei L, Montaño R. Binational population of jaguars confirmed by camera-trapping in the American Gran Chaco. Cat News. 2007; 46: 24-25.
19. Montaño R, Maffei L, Noss A. Segundo muestreo con trampas cámaras de jaguares y otros mamíferos en el Campamento Palmar de las Islas y Ravelo. Diciembre 2006-Marzo 2007. Santa Cruz, Bolivia, Wildlife Conservation Society 2010; Technical Report #185.
20. Romero-Muñoz A, Montaño R, Peña R, Dosapei T, Paredes R. Muestreo con trampas-cámara de jaguares y otros mamíferos en el Campamento Palmar de las Islas. Santa Cruz, Bolivia, Wildlife Conservation Society 2006; Technical Report #167.
21. Romero-Muñoz A. Densidad, patrones de actividad y comportamiento espacial de felinos en dos sitios del Gran Chaco con diferente presión de ganadería: Universidad Mayor de San Simón; 2008.
22. Cuéllar E, Dosapei T, Peña R, Noss A. Jaguar and other mammal camera trap survey Ravelo field camp (19° 17' 44" S, 60° 37' 10" W). Kaa-Iya del Gran Chaco National Park, 7 February-9 April 2003. Santa Cruz, Bolivia, CABI, Wildlife Conservation Society 2003; Technical Report #91.
23. Cuéllar E, Dosapei T, Peña R, Noss A. Jaguar and other mammal camera trap survey Ravelo II, Ravelo field camp (19° 17' 44" S, 60° 37' 10" W). Kaa-Iya del Gran Chaco National Park, 18 September-18 November 2003. Santa Cruz, Bolivia, Wildlife Conservation Society 2003; Technical Report #103.
24. Peña R, Dosapei T, Cuéllar E. Densidad y área mínima de acción del jaguar (*Panthera onca*) en dos épocas del año en Ravelo, Parque Nacional Kaa-Iya, Santa Cruz, Bolivia. Memorias: Manejo de Fauna Silvestre en la Amazonía y Latinoamérica. 2004; 4:248-250. [www.revistafauna.com.pe/memo.htm](http://www.revistafauna.com.pe/memo.htm).
25. Maffei L, Cuéllar E, Peña R, Dosapei T, Julio B, Noss A. Jaguar and other mammal camera trap survey, Tucavaca field camp (18° 30.97’ S, 60° 48.62’ W). Kaa-Iya del Gran Chaco National Park, 15 January-20 March 2002. Santa Cruz, Bolivia, CABI, Wildlife Conservation Society 2002; Technical Report #83.
26. Maffei L, Julio B, Noss A. Jaguar and other mammal camera trap survey Tucavaca II, Tucavaca field camp (18° 30.97’ S, 60° 48.62’ W). Kaa-Iya del Gran Chaco National Park, 12 April-12 June 2003. Santa Cruz, Bolivia, CABI, Wildlife Conservation Society 2003; Technical Report #97.
27. Maffei L, Julio B, Paredes R, Posiño A, Noss A. J. Estudios con trampas-cámara en el campamento Tucavaca III (18° 30.97’ S, 60° 48.62’ W). Parque Nacional Kaa-Iya del Gran Chaco, 28 de marzo-28 de mayo de 2004. Santa Cruz, Bolivia, Wildlife Conservation Society 2004; Technical Report #129.
28. Maffei L, Paredes R, Aguanta F, Noss A. Muestreo con trampas-cámara de jaguares y otros mamíferos en la Estación Isoso (18° 25’ S, 61° 46’ W). Parque Nacional Kaa-Iya del Gran Chaco, 28 de Octubre-24 de Diciembre 2005. Santa Cruz, Bolivia, Wildlife Conservation Society 2006; Technical Report #161.
29. Romero-Muñoz A, Paredes R, Maffei L. Muestreo con trampas cámara de jaguares y otros mamíferos en la Estación Isoso (18°25’W, 61°46’W). Parque Nacional Kaa Iya del Gran Chaco. 7 de Julio – 8 de septiembre, 2006. Santa Cruz, Bolivia, Wildlife Conservation Society 2007; Technical Report #172.
30. Arispe R, Rumiz DI, Venegas C. Segundo censo de jaguares (*Panthera onca*) y otros mamíferos con trampas cámara en la Estancia San Miguelito. Santa Cruz, Bolivia, Wildlife Conservation Society 2005, Technical Report #144.
31. Arispe R, Rumiz DI, Venegas C. Censo de jaguares (*Panthera onca*) y otros mamíferos con trampas-cámara en la Concesión Forestal El Encanto (23 de septiembre–20 de noviembre 2006). Santa Cruz, Bolivia, Wildlife Conservation Society 2007; Technical Report #173.
32. Wallace RB, Gomez H, Ayala G, Espinoza F. Camera trapping for jaguar (*Panthera onca*) in the Tuichi Valley, Bolivia. Mastozool Neotrop. 2003; 10: 133-139.
33. Cullen Jr L. Jaguar as landscape detectives for the conservation in the Atlantic Forest of Brazil. Canterbury, UK: University of Kent; 2006.
34. Soisalo MK, Cavalcanti SMC. Estimating the density of a jaguar population in the Brazilian Pantanal using camera-traps and capture-recapture sampling in combination with GPS radio-telemetry. Biol Conserv. 2006; 129: 487-496.
35. Silveira L. Ecologia comparada e conservação da onça-pintada (*Panthera onca*) e onça-parda (*Puma concolor*), no Cerrado e Pantanal. Brasilia: Universidade de Brasília; 2004.
36. Sollmann R, Furtado MM, Gardner B, Hofer H, Jácomo ATA, Tôrres NM, Silveria L. Improving density estimates for elusive carnivores: Accounting for sex-specific detection and movements using spatial capture–recapture models for jaguars in central Brazil. Biol Conserv. 2011; 144: 1017-1024. doi:http://dx.doi.org/10.1016/j.biocon.2010.12.011
37. Negrões N, Sollmann R, Fonseca C, Jácomo A, Revilla E, Silveira L. One or two cameras per station? Monitoring jaguars and other mammals in the Amazon. Ecol Res. 2012; 27: 639-648. doi:10.1007/s11284-012-0938-4
38. Silveira L, Jácomo ATA, Astete S, Sollmann R, Tôrres NM, Furtado MM, Marinho-Filho J. Density of the Near Threatened jaguar *Panthera onca* in the caatinga of north-eastern Brazil. Oryx. 2010; 44: 104-109. doi:10.1017/S0030605309990433
39. Sollmann R, Tôrres NM, Furtado MM, de Almeida Jácomo AT, Palomares F, Roques S, Silveira L. Combining camera-trapping and noninvasive genetic data in a spatial capture–recapture framework improves density estimates for the jaguar. Biol Conserv. 2013; 167: 242-247. doi:<http://dx.doi.org/10.1016/j.biocon.2013.08.003>
40. Payán E. Hunting sustainability, species richness and carnivore conservation in Colombian Amazonia. London: London University College; 2009.
41. Boron V, Tzanopoulos J, Gallo J, Barragan J, Jaimes-Rodriguez L, Schaller G, Payán E. Jaguar Densities across Human-Dominated Landscapes in Colombia: The Contribution of Unprotected Areas to Long Term Conservation. PLoS ONE. 2016; 11: e0153973.
42. Bustamante A. Densidad y uso de hábitat por los felinos en la parte sureste del área de amortiguamiento del Parque Nacional Corcovado, Península de Osa, Costa Rica. Universidad Nacional Heredia, Costa Rica. 2008.
43. Salom-Pérez R, Carrillo E, Sáenz JC, Mora JM. Critical condition of the jaguar *Panthera onca* population in Corcovado National Park, Costa Rica. Oryx. 2007; 41: 51-56. doi:10.1017/S0030605307001615
44. Amit R, Alfaro L, Carrillo E. Estimación de poblaciones de jaguar (*Panthera onca*) en el Área de Conservación Guanacaste, Costa Rica. Ambientales. 2009; 38: 3-5.
45. Araguillín E, Ríos G Z, Utreras V, Noss A. Muestreo con trampas fotográficas de mamíferos medianos, grandes y de aves en el Bloque Ishpingo Tambococha Tiputini (ITT), sector Varadero (Parque Nacional Yasuní) 2010; Informe Técnico 9.
46. García A, McNab R, Soto J, Radachowsky J, Moreira J, Estrada C, Méndez V, Juárez D, Dubón T, Córdova M. Los jaguares del corazón del Parque Nacional Tikal, Petén, Guatemala. Asociación Balám and Wildlife Conservation Society, Flores, Petén, Guatemala. 2006. <https://library.wcs.org/doi/ctl/view/mid/33065/pubid/DMX960100000.aspx>
47. Moreira J, McNab R, García R, Ponce-Santizo G, Mérida M, Méndez V, Córdova M, Ruano G, Tut K, Tut H, Córdova F, Muñoz E, González E, Cholom J, Xol A. Abundancia y densidad de Jaguares en el Parque Nacional Laguna del Tigre-Corredor Biológico Central, Reserva de la Biosfera Maya. Informe Interno WCS-Programa para Guatemala. 2009; 35. <https://guatemala.wcs.org/DesktopModules/Bring2mind/DMX/Download.aspx?EntryId=9618&PortalId=115&DownloadMethod=attachment>.
48. Moreira J, García R, McNab R, Ponce-Santizo G, Mérida M, Ruano G, Méndez V. Abundancia de Jaguares y Evaluación de Presas Asociadas al Fototrampeo en las Concesiones Comunitarias del Bloque de Melchor de Mencos, Reserva de la Biosfera Maya, Petén, Guatemala. Informe Interno WCS-Programa para Guatemala. 2010; 55. <https://guatemala.wcs.org/DesktopModules/Bring2mind/DMX/Download.aspx?EntryId=9571&PortalId=115&DownloadMethod=attachment>
49. Moreira J, McNab, García R, Méndez V, Barnes M, Ponce G, Vanegas A, Ical G, Zepeda E, García I. Densidad de jaguares dentro de la concesión comunitaria de Carmelita y de la Asociación Forestal Integral San Andrés Petén. Guatemala, 2008. <https://library.wcs.org/doi/ctl/view/mid/33065/pubid/DMX957300000.aspx>
50. Moreira J, Balas R, Thornton D, García R, Méndez V, Vanegas A, Ical G, Zepeda E, Senturión R, García I. Abundancia de jaguares en La Gloria-El Lechugal, Zona de Usos Múltiples, Reserva de la Biosfera Maya, Petén, Guatemala. Sociedad para la Conservación de la Vida Silvestre (WCS-Guatemala), Programa para la Conservación del Jaguar 2007. <https://guatemala.wcs.org/DesktopModules/Bring2mind/DMX/Download.aspx?EntryId=9638&PortalId=115&DownloadMethod=attachment>.
51. Moreira J, García R, McNab RB, Ruano G, Ponce G, Mérida M, Tut K, Díaz P, González E, Córdova M, Centeno E, López C, Vanegas A, Vanegas Y, Córdova F, Kay J, Polanco G, Barnes M. Abundancia de Jaguares y Presas Asociadas al Fototrampeo en el sector oeste del Parque Nacional Mirador - Río Azul, Reserva de Biosfera Maya. Wildlife Conservation Society, 2011. http://www.chmguatemala.gob.gt/images/biodiversidad/conservacion/Abundancia%20de%20Jaguares%20y%20Presas%20Asociadas%20al%20fototrampeo%20en%20el%20sector%20oeste%20del%20parque%20ancional%20mirador%20rio%20azul%20Moreira%20et%20al.%202011_Jaguares%20Mirador.pdf131
52. Moreira J, McNab R, García R, Méndez V, Ponce-Santizo G, Córdova M, Tun S, Caal T, Corado J. Densidad de jaguares en el Biotopo Protegido Dos Lagunas, Parque Nacional Mirador Río Azul, Petén, Guatemala. Informe Interno WCS-Programa para Guatemala 2008.
53. Association Kwata, Etude des carnivores sur la réserve naturelle nationale des Nouragues le jaguar. 2013. <http://www.kwata.net/medias/images/upload/Rapport%20DEAL_Carnivores%20_2012.pdf>.
54. de Thoisy B, Poirier E. Jaguar densities in French Guiana. Jaguar News. 2009; 28.
55. De Thoisy B, Fayad I, Clément L, Barrioz S, Poirier E, Gond V. Predators, prey and habitat structure: can key conservation areas and early signs of population collapse be detected in neotropical forests? PLoS ONE. 2016; 11: e0165362.
56. Paemelaere E A D. The Panthera Jaguar Corridor Initiative: Wildlife populations of the Rupununi - an assessment of relative abundance, Karanambu – Dadanawa. Finall report 2011-2012, EPA Reference No: 220311 BR155 2013.
57. Paemelaere E A D, Payán E. Jaguar and Prey Populations within Human Dominated Landscapes in Guyana: Logging Concessions. Panthera Report 2013
58. de la Torre JA, Medellín RA. Jaguars *Panthera onca* in the Greater Lacandona Ecosystem, Chiapas, Mexico: population estimates and future prospects. Oryx. 2011; 45: 546-553. doi:10.1017/S0030605310001511
59. Núñez-Pérez R. Estimating jaguar population density using camera-traps: a comparison with radio-telemetry estimates. J Zool. 2011; 285: 39-45. doi:10.1111/j.1469-7998.2011.00812.x
60. Coronel Arellano H, López González CA, Lorenzana Piña G, Ortega Huerta MA. El jaguar (*Panthera onca*) en Queretaro. Extensión Nuevos Tiempos. 2008; 2: 29-34.
61. Ávila-Nájera DM, Chávez C, Lazcano-Barrero MA, Pérez-Elizalde S, Alcántara-Carbajal JL. Estimación poblacional y conservación de felinos (Carnivora: Felidae) en el norte de Quintana Roo, México. Rev Biol Trop. 2015; 63: 799-813.
62. Faller JC, Chávez C, Johnson S, Ceballos G. Densidad y tamaño de la población de jaguar en el noreste de la Península de Yucatán. In: Ceballos G, Chávez C, List R, Zarza E, editors. Conservación y manejo del jaguar en México: estudios de caso y perspectivas. México: Conabio- Alianza WWF/Telcel- Universidad Nacional Autónoma de México; 2007. p. 111-122.
63. Reserva de la Biosfera Marismas Nacionales. Monitoreo de jaguar (*Panthera onca*) en la reserva de la Biosfera Marismas Nacionales. Comisión Nacional de Áreas Naturales Protegidas, México, D.F.2011
64. Avila Nájera MD. Abundancia del jaguar (*Panthera onca*) y de sus presas en el municipio de Tamasopo, San Luis Potosí. Montecillo, Mexico: Instituto de enseñanza e investigación en ciencias agricolas; 2009.
65. Carrera-Treviño R, Lira-Torres I, Martínez-García L, López-Hernández M. El jaguar *Panthera onca* (Carnivora: Felidae) en la Reserva de la Biosfera “El Cielo”, Tamaulipas, México. Rev Biol Trop. 2016; 64: 1451-1468.
66. Rosas-Rosas OC, Bender LC. Estado de la población de jaguares (*Panthera onca*) y pumas (*Puma concolor*) en el noreste de Sonora, México. Acta Zoológica Mexicana. 2012; 28: 86-101.
67. Gutiérrez-González CE, Gómez-Ramírez MÁ, López-González CA. Estimation of the density of the Near Threatened jaguar *Panthera onca* in Sonora, Mexico, using camera trapping and an open population model. Oryx. 2012; 46: 431-437. doi:10.1017/S003060531100041X
68. Moreno R. Parámetros poblacionales y aspectos ecológicos de los felinos y sus presas en Cana, Parque Nacional Darién, Panamá. Instituto Internacional en conservación y Manejo de Vida Silvestre, Heredia, Costa Rica; 2006.
69. Tobler MW, Carrillo-Percastegui SE, Zúñiga Hartley A, Powell GVN. High jaguar densities and large population sizes in the core habitat of the southwestern Amazon. Biol Conserv. 2013; 159: 375-381. doi:http://dx.doi.org/10.1016/j.biocon.2012.12.012
70. Jędrzejewski W, Puerto MF, Goldberg JF, Hebblewhite M, Abarca M, Gamarra G, Calderón LE, Romero JF, Viloria ÁL, Carreño R. Density and population structure of the jaguar (*Panthera onca*) in a protected area of Los Llanos, Venezuela, from 1 year of camera trap monitoring. Mammal Research. 2017; 62: 9-19. doi:10.1007/s13364-016-0300-2
