# Supplementary material for: Estimating large carnivore populations at global scale based on spatial predictions of density and distribution – Application to the jaguar (Panthera onca)
Source: PLoS One. 2018 Mar 26;13(3):e0194719. doi: 10.1371/journal.pone.0194719 (PMC5868828; doi:10.1371/journal.pone.0194719)
Supplement: S2 Table — (DOCX) [file pone.0194719.s002.docx]

**Estimating large carnivore populations at global scale based on spatial predictions of density and distribution – application to the jaguar (*Panthera onca*)**

Jędrzejewski W.*, Robinson H.S., Abarca M., Zeller K.A., Velasquez G., Paemelaere E.A.D., Goldberg J.F., Payan E., Hoogesteijn R., Boede E.O., Schmidt K., Lampo M., Viloria Á.L., Carreño R., Robinson N., Lukacs P.M., Nowak J.J., Salom-Pérez R., Castañeda F., Boron V., Quigley H.

*correspondence to: [wjedrzej1@gmail.com](file:///C:\MDoc-Venezuela-S\Papers-manuscripts\Jaguar_Americas_Distr_Dens_Numb_2\PlosBiology\wjedrzej1@gmail.com)

**S2 Table. Candidate predictive variables used in the spatial analysis**.

| **No** | **Variable code** | **Full variable name and units** | **Data description / Time period to which the data refer/Notes** | **Source** | **Reference #** |
| --- | --- | --- | --- | --- | --- |
| 1 | TEMP | Mean annual temperature (^o^C) | Mean from 1950 - 2000;  The original value downloaded from the WorldClim webpage was divided by 10 to express it in Centi-grades. | Bioclim WorldClim - Global Climate Data www.worldclim.org/bioclim | [1] |
| 2 | PREC | Annual precipitation  (mm) | In the tropics precipitation values are usually related to productivity  Mean from 1950 - 2000 | Bioclim WorldClim - Global Climate Data  www.worldclim.org/bioclim | [1] |
| 3 | CANOPY | Mean forest canopy cover (%) | Mean from 2000 - 2011 | MODIS: MOD44B  https://lpdaac.usgs.gov/ | [2,3] |
| 4 | NPP_MEAN_ | Mean net primary productivity  (grams of elemental carbon per m^2^) | Raster with the net amount of solar energy converted to plant organic matter through photosynthesis  Mean from 1981-2014 | MODIS: MOD17A3  http://www.ntsg.umt.edu/project/mod17 | [4] |
| 5 | NPP_SD_ | Standard deviation of net primary productivity | May indicate seasonal variation or seasonal abundance of plant organic matter  Mean from 1981-2014 | MODIS: MOD17A3  http://www.ntsg.umt.edu/project/mod17 | [4] |
| 6 | GPP_MEAN_ | Mean gross primary productivity (grams of elemental carbon per m^2^) | Raster with the net amount of solar energy converted to plant organic matter through photosynthesis.  Mean from 1981-2014 | MODIS: MOD17A3  http://www.ntsg.umt.edu/project/mod17 | [4] |
| 7 | GPP_SD_ | Standard deviation of gross primary productivity | May indicate seasonal variation or seasonal abundance of plant organic matter  Mean from 1981-2014 | MODIS: MOD17A3  http://www.ntsg.umt.edu/project/mod17 | [4] |
| 8 | NDVI_MEAN_ | Mean normalized difference vegetation index | Vegetation productivity measure  Mean from 1981-2014 | MODIS:MCD43A4_NDVI  https://lpdaac.usgs.gov/ | [5,6] |
| 9 | NDVI_SD_ | Standard deviation of normalized difference vegetation index | Measure of variability/seasonality of vegetation  Mean from 1981-2014 | MODIS:MCD43A4_NDVI  https://lpdaac.usgs.gov/ | [5,6] |
| 10 | EVI_MEAN_ | Enhanced vegetation index | Vegetation productivity measure  Mean from 1981-2014 | MODIS:MCD43A4_EVI  https://lpdaac.usgs.gov/ | [7-9] |
| 11 | EVI_SD_ | Standard deviation of enhanced vegetation index | Measure of variability/seasonality of vegetation  Mean from 1981-2014 | MODIS:MCD43A4_EVI  https://lpdaac.usgs.gov/ | [7-9] |
| 12 | NDWI_MEAN_ | Mean annual value of normalized difference water index (values -1 to 1) | Measure of ground water abundance; positive data values are typically open water areas; while the negative values are typically non-water features (i.e. terrestrial vegetation and bare soil)  Mean from 1981-2014 | MODIS:MCD43A4_NDWI  https://lpdaac.usgs.gov/ | [10] |
| 13 | NDWI_SD_ | Standard deviation of normalized difference water index | Variability/seasonality in water abundance; high values may indicate regular flooding or seasonal abundance of water  Mean from 1981-2014 | MODIS:MCD43A4_NDWI  https://lpdaac.usgs.gov/… | [10] |
| 14 | HPDENLG | Human population density people/km^2^, logarithmically transformed | Data for 2011 | http://sedac.ciesin.columbia.edu/data | [11] |
| 15 | HFOOTP | Human footprint index IGHP, V2, 1995-2004 (values 0 – 100) | Index reflecting human caused environmental changes  Data from 1995 - 2004 | NASA Socioeconomic Data and Applications Center (SEDAC) <http://sedac.ciesin.columbia.edu/data> | [12] |
| 16 | PRAR | Protection status: if inside a protected area -value 1, if not – value 0 | World map of protected areas. We added Hato Piñero and Hato El Frío as known jaguar protected areas in Venezuela  Data for 2015 | http://www.protectedplanet.net/ | [13] |
| 17 | NA-SA | Distinction between North and South America (code 1 for North America and 2 for South America |  |  |  |

**References for S2 Table:**

1. Hijmans RJ, Cameron SE, Parra JL, Jones PG, Jarvis A. Very high resolution interpolated climate surfaces for global land areas. Int J Climatol*.*2005; 25: 1965-1978. doi:10.1002/joc.1276
2. Hansen M, DeFries R, Townshend J, Carroll M, Dimiceli C, Sohlberg R. Global percent tree cover at a spatial resolution of 500 meters: First results of the MODIS vegetation continuous fields algorithm. Earth Interact. 2003; 7: 1-15.
3. DiMiceli C, Carroll M, Sohlberg R, Huang C, Hansen M, Townshend J. Annual global automated MODIS vegetation continuous fields (MOD44B) at 250 m spatial resolution for data years beginning day 65, 2000–2010, collection 5 percent tree cover. University of Maryland, College Park, MD, USA. 2011.
4. Zhao M, Heinsch FA, Nemani RR, Running SW. Improvements of the MODIS terrestrial gross and net primary production global data set. Remote Sens Environ. 2005; 95: 164-76. doi:10.1016/j.rse.2004.12.011
5. Pettorelli N, Ryan S, Mueller T, Bunnefeld N, Jedrzejewska B, Lima M, Kausrud K. The Normalized Difference Vegetation Index (NDVI): unforeseen successes in animal ecology. Clim Res. 2011; 46: 15-27. doi:10.3354/cr00936
6. Pettorelli N, Vik JO, Mysterud A, Gaillard J-M, Tucker CJ, Stenseth NC. Using the satellite-derived NDVI to assess ecological responses to environmental change. Trends Ecol Evol. 2005; 20: 503-10.
7. Huete A, Didan K, Miura T, Rodriguez EP, Gao X, Ferreira LG. Overview of the radiometric and biophysical performance of the MODIS vegetation indices. Remote Sens Environ. 2002; 83: 195-213.
8. Huete AR, Didan K, Shimabukuro YE, Ratana P, Saleska SR, Hutyra LR, Yang W, Nemani RR, Myneni R. Amazon rainforests green‐up with sunlight in dry season. Geophysical Research Letters. 2006; 33. doi:10.1029/2005gl025583
9. Jiang Z, Huete AR, Didan K, Miura T. Development of a two-band enhanced vegetation index without a blue band. Remote Sens Environ. 2008; 112: 3833-45. doi:10.1016/j.rse.2008.06.006
10. McFeeters S. The use of the Normalized Difference Water Index (NDWI) in the delineation of open water features. International journal of remote sensing. 1996; 17: 1425-32.
11. Balk D, Deichmann U, Yetman G, Pozzi F, Hay S, Nelson A. Determining global population distribution: methods, applications and data. Adv Parasitol. 2006; 62: 119-56.
12. Sanderson EW, Jaiteh M, Levy MA, Redford KH, Wannebo AV, Woolmer G. The Human Footprint and the Last of the Wild The human footprint is a global map of human influence on the land surface, which suggests that human beings are stewards of nature, whether we like it or not. Bioscience. 2002; 52: 891-904. doi: [http://dx.doi.org/10.1641/0006-3568(2002)052[0891:THFATL]2.0.CO;2](http://dx.doi.org/10.1641/0006-3568(2002)052%5b0891:THFATL%5d2.0.CO;2)
13. IUCN & UNEP-WCMC. The World Database on Protected Areas (WDPA) [On-line], [2015], Cambridge, UK: UNEP-WCMC. Available at: www.protectedplanet.net. (2016)
