# Supplementary material for: Estimating large carnivore populations at global scale based on spatial predictions of density and distribution – Application to the jaguar (Panthera onca)
Source: PLoS One. 2018 Mar 26;13(3):e0194719. doi: 10.1371/journal.pone.0194719 (PMC5868828; doi:10.1371/journal.pone.0194719)
Supplement: S3 Table — (DOCX) [file pone.0194719.s003.docx]

**Estimating large carnivore populations at global scale based on spatial predictions of density and distribution – application to the jaguar (*Panthera onca*)**

Jędrzejewski W.*, Robinson H.S., Abarca M., Zeller K.A., Velasquez G., Paemelaere E.A.D., Goldberg J.F., Payan E., Hoogesteijn R., Boede E.O., Schmidt K., Lampo M., Viloria Á.L., Carreño R., Robinson N., Lukacs P.M., Nowak J.J., Salom-Pérez R., Castañeda F., Boron V., Quigley H.

*correspondence to: [wjedrzej1@gmail.com](file:///C:\\MDoc-Venezuela-S\\Papers-manuscripts\\Jaguar_Americas_Distr_Dens_Numb_2\\PlosBiology\\wjedrzej1@gmail.com)

**S3 Table. Pearson correlation matrix for jaguar density estimates (dependent variable) and a set of predictive variables.** Density estimates (JDen_SCR) were obtained with spatial capture-recapture or transformed to spatial capture-recapture level (see Methods). Abbreviations for the predictive environmental variables are explained in S2 Table. For density estimates and study effort variables see S1 Table. Variables marked with ® were not entered to the regression analyses due to high correlation with other variables (r > 0.7).

|  | JDen_SCR | TEMP | PREC | NPPmean | NPP_SD_ | GPP_mean_ ® | GPP_SD_ | EVI_mean_ | EVI_SD_ | NDVI_mean_® | NDVI_SD_® | NDWI_mean_® | NDWI_SD_ ® | CANOPY® | HPDENLG | HFOOTP | PRAR | NA-SA | N STATIONS | N STUDY DAYS | POLYGON SIZE | TRAP NIGHTS® |
| --- | --- | --- | --- | --- | --- | --- | --- | --- | --- | --- | --- | --- | --- | --- | --- | --- | --- | --- | --- | --- | --- | --- |
| JDen_SCR | 1.00 |  |  |  |  |  |  |  |  |  |  |  |  |  |  |  |  |  |  |  |  |  |
| TEMP | 0.47 | 1.00 |  |  |  |  |  |  |  |  |  |  |  |  |  |  |  |  |  |  |  |  |
| PREC | 0.30 | 0.23 | 1.00 |  |  |  |  |  |  |  |  |  |  |  |  |  |  |  |  |  |  |  |
| NPP_MEAN_ | 0.24 | -0.25 | 0.36 | 1.00 |  |  |  |  |  |  |  |  |  |  |  |  |  |  |  |  |  |  |
| NPP_SD_ | 0.13 | 0.01 | -0.15 | -0.10 | 1.00 |  |  |  |  |  |  |  |  |  |  |  |  |  |  |  |  |  |
| GPP_MEAN_ ® | 0.37 | -0.06 | 0.44 | 0.90 | -0.06 | 1.00 |  |  |  |  |  |  |  |  |  |  |  |  |  |  |  |  |
| GPP_SD_ | -0.06 | -0.22 | 0.04 | 0.15 | 0.36 | 0.16 | 1.00 |  |  |  |  |  |  |  |  |  |  |  |  |  |  |  |
| EVI_MEAN_ | 0.47 | 0.17 | 0.57 | 0.52 | 0.10 | 0.77 | 0.12 | 1.00 |  |  |  |  |  |  |  |  |  |  |  |  |  |  |
| EVI_SD_ | -0.19 | -0.17 | -0.51 | -0.43 | 0.28 | -0.50 | 0.28 | -0.40 | 1.00 |  |  |  |  |  |  |  |  |  |  |  |  |  |
| NDVI_MEAN_ ® | 0.30 | -0.09 | 0.47 | 0.63 | 0.20 | 0.79 | 0.23 | 0.91 | -0.40 | 1.00 |  |  |  |  |  |  |  |  |  |  |  |  |
| NDVI_SD_ ® | -0.13 | 0.15 | -0.39 | -0.66 | 0.00 | -0.74 | 0.07 | -0.64 | 0.73 | -0.75 | 1.00 |  |  |  |  |  |  |  |  |  |  |  |
| NDWI_MEAN_ ® | 0.30 | 0.09 | 0.52 | 0.48 | -0.10 | 0.65 | 0.09 | 0.77 | -0.50 | 0.75 | -0.61 | 1.00 |  |  |  |  |  |  |  |  |  |  |
| NDWI_SD_ ® | -0.17 | 0.06 | -0.56 | -0.50 | 0.06 | -0.63 | 0.07 | -0.68 | 0.79 | -0.70 | 0.82 | -0.62 | 1.00 |  |  |  |  |  |  |  |  |  |
| CANOPY® | 0.34 | -0.11 | 0.39 | 0.57 | 0.13 | 0.73 | 0.14 | 0.82 | -0.43 | 0.90 | -0.66 | 0.73 | -0.71 | 1.00 |  |  |  |  |  |  |  |  |
| HPDENLG | -0.03 | -0.22 | 0.00 | 0.27 | -0.13 | 0.27 | 0.14 | 0.12 | -0.10 | 0.22 | -0.24 | 0.25 | -0.10 | 0.16 | 1.00 |  |  |  |  |  |  |  |
| HFOOTP | -0.12 | -0.25 | 0.04 | 0.18 | -0.23 | 0.15 | 0.25 | 0.05 | 0.04 | 0.11 | -0.13 | 0.19 | 0.04 | -0.03 | 0.68 | 1.00 |  |  |  |  |  |  |
| PRAR | -0.05 | -0.26 | 0.10 | 0.26 | 0.12 | 0.28 | 0.11 | 0.28 | -0.03 | 0.37 | -0.28 | 0.37 | -0.15 | 0.30 | 0.27 | 0.24 | 1.00 |  |  |  |  |  |
| NA-SA | -0.31 | -0.16 | 0.04 | 0.01 | 0.27 | -0.17 | 0.22 | -0.22 | 0.09 | -0.03 | 0.02 | -0.26 | 0.09 | -0.11 | -0.34 | -0.30 | -0.13 | 1.00 |  |  |  |  |
| N STATIONS | -0.17 | 0.03 | 0.13 | -0.03 | 0.00 | -0.12 | -0.11 | -0.03 | 0.06 | -0.05 | -0.04 | -0.05 | 0.02 | -0.21 | -0.07 | 0.12 | -0.04 | 0.11 | 1.00 |  |  |  |
| N STUDY DAYS | -0.10 | -0.28 | -0.14 | -0.03 | -0.10 | -0.12 | -0.11 | -0.24 | 0.14 | -0.21 | 0.18 | -0.24 | 0.13 | -0.09 | 0.05 | 0.09 | -0.07 | -0.07 | 0.02 | 1.00 |  |  |
| POLYGON SIZE | -0.35 | -0.45 | -0.11 | 0.00 | 0.10 | -0.11 | 0.19 | -0.14 | 0.26 | -0.03 | 0.00 | -0.13 | 0.07 | -0.06 | -0.05 | 0.09 | -0.01 | 0.29 | 0.35 | 0.07 | 1.00 |  |
| TRAP NIGHTS® | -0.14 | -0.27 | -0.03 | 0.11 | -0.02 | -0.02 | -0.09 | -0.12 | 0.08 | -0.02 | -0.02 | -0.14 | 0.00 | -0.01 | 0.10 | 0.14 | -0.02 | 0.08 | 0.46 | 0.80 | 0.22 | 1.00 |
