# Supplementary material for: Estimating large carnivore populations at global scale based on spatial predictions of density and distribution – Application to the jaguar (Panthera onca)
Source: PLoS One. 2018 Mar 26;13(3):e0194719. doi: 10.1371/journal.pone.0194719 (PMC5868828; doi:10.1371/journal.pone.0194719)
Supplement: S4 Table — (DOCX) [file pone.0194719.s004.docx]

**Estimating large carnivore populations at global scale based on spatial predictions of density and distribution – application to the jaguar (*Panthera onca*)**

Jędrzejewski W.*, Robinson H.S., Abarca M., Zeller K.A., Velasquez G., Paemelaere E.A.D., Goldberg J.F., Payan E., Hoogesteijn R., Boede E.O., Schmidt K., Lampo M., Viloria Á.L., Carreño R., Robinson N., Lukacs P.M., Nowak J.J., Salom-Pérez R., Castañeda F., Boron V., Quigley H.

*correspondence to: [wjedrzej1@gmail.com](file:///C:\\MDoc-Venezuela-S\\Papers-manuscripts\\Jaguar_Americas_Distr_Dens_Numb_2\\PlosBiology\\wjedrzej1@gmail.com)

**S4 Table. Pearson correlation matrix for the jaguar occurrence (dependent variable) and a set of predictive variables.** Jaguar occurrence (JOcc) is based on presence/absence data (S12 Data). Abbreviations for the predictive environmental variables are explained in S2 Table. Variables marked with ® were not entered to the logistic regression analyses due to high correlation with other variables (r > 0.7).

|  | JOcc | TEMP | PREC | NPPmean | NPP_SD_ | GPP_mean_® | GPP_SD_ | EVI_mean_® | EVI_SD_® | NDVI_mean_® | NDVI_SD_ | NDWI_mean_ | NDWI_SD_® | CANOPY | HPDENLG | HFOOTP | PRAR | NA-SA |
| --- | --- | --- | --- | --- | --- | --- | --- | --- | --- | --- | --- | --- | --- | --- | --- | --- | --- | --- |
| JOcc | 1.00 |  |  |  |  |  |  |  |  |  |  |  |  |  |  |  |  |  |
| TEMP | 0.38 | 1.00 |  |  |  |  |  |  |  |  |  |  |  |  |  |  |  |  |
| **PREC** | 0.42 | 0.33 | 1.00 |  |  |  |  |  |  |  |  |  |  |  |  |  |  |  |
| NPP_MEAN_ | 0.30 | 0.12 | 0.45 | 1.00 |  |  |  |  |  |  |  |  |  |  |  |  |  |  |
| NPP_SD_ | 0.13 | 0.18 | 0.04 | 0.20 | 1.00 |  |  |  |  |  |  |  |  |  |  |  |  |  |
| GPP_MEAN_ ® | 0.41 | 0.28 | 0.54 | 0.90 | 0.30 | 1.00 |  |  |  |  |  |  |  |  |  |  |  |  |
| GPP_SD_ | 0.14 | 0.06 | 0.08 | 0.51 | 0.52 | 0.47 | 1.00 |  |  |  |  |  |  |  |  |  |  |  |
| EVI_MEAN_® | 0.42 | 0.44 | 0.54 | 0.65 | 0.32 | 0.83 | 0.39 | 1.00 |  |  |  |  |  |  |  |  |  |  |
| EVI_SD_® | -0.11 | 0.11 | -0.25 | -0.05 | 0.21 | -0.08 | 0.51 | 0.07 | 1.00 |  |  |  |  |  |  |  |  |  |
| NDVI_MEAN_ ® | 0.46 | 0.40 | 0.56 | 0.66 | 0.37 | 0.82 | 0.46 | 0.94 | 0.07 | 1.00 |  |  |  |  |  |  |  |  |
| NDVI_SD_ | -0.15 | 0.09 | -0.26 | -0.26 | 0.11 | -0.29 | 0.33 | -0.17 | 0.85 | -0.17 | 1.00 |  |  |  |  |  |  |  |
| NDWI_MEAN_ | 0.38 | 0.30 | 0.50 | 0.52 | 0.12 | 0.67 | 0.13 | 0.71 | -0.17 | 0.64 | -0.32 | 1.00 |  |  |  |  |  |  |
| NDWI_SD_ ® | -0.12 | 0.21 | -0.26 | -0.24 | 0.07 | -0.30 | 0.22 | -0.20 | 0.76 | -0.16 | 0.77 | -0.26 | 1.00 |  |  |  |  |  |
| CANOPY | 0.52 | 0.21 | 0.52 | 0.62 | 0.22 | 0.77 | 0.33 | 0.74 | -0.16 | 0.78 | -0.31 | 0.70 | -0.35 | 1.00 |  |  |  |  |
| HPDENLG | -0.30 | -0.09 | -0.03 | 0.06 | -0.03 | 0.08 | -0.04 | 0.12 | 0.13 | 0.07 | 0.03 | 0.13 | 0.04 | -0.05 | 1.00 |  |  |  |
| HFOOTP | -0.36 | -0.05 | -0.11 | -0.05 | -0.10 | -0.07 | -0.06 | -0.01 | 0.22 | -0.08 | 0.14 | 0.02 | 0.18 | -0.24 | 0.69 | 1.00 |  |  |
| PRAR | 0.34 | 0.05 | 0.28 | 0.26 | 0.00 | 0.32 | 0.07 | 0.28 | -0.19 | 0.28 | -0.18 | 0.31 | -0.23 | 0.40 | -0.08 | -0.22 | 1.00 |  |
| NA-SA | -0.03 | 0.10 | -0.07 | 0.04 | 0.24 | -0.01 | 0.14 | 0.07 | 0.10 | 0.13 | 0.07 | -0.06 | 0.13 | -0.04 | -0.12 | -0.11 | -0.10 | 1.00 |
