# Supplementary material for: Estimating large carnivore populations at global scale based on spatial predictions of density and distribution – Application to the jaguar (Panthera onca)
Source: PLoS One. 2018 Mar 26;13(3):e0194719. doi: 10.1371/journal.pone.0194719 (PMC5868828; doi:10.1371/journal.pone.0194719)
Supplement: S6 Table — (DOCX) [file pone.0194719.s006.docx]

**Estimating large carnivore populations at global scale based on spatial predictions of density and distribution – application to the jaguar (*Panthera onca*)**

Jędrzejewski W.*, Robinson H.S., Abarca M., Zeller K.A., Velasquez G., Paemelaere E.A.D., Goldberg J.F., Payan E., Hoogesteijn R., Boede E.O., Schmidt K., Lampo M., Viloria Á.L., Carreño R., Robinson N., Lukacs P.M., Nowak J.J., Salom-Pérez R., Castañeda F., Boron V., Quigley H.

*correspondence to: [wjedrzej1@gmail.com](file:///C:\MDoc-Venezuela-S\Papers-manuscripts\Jaguar_Americas_Distr_Dens_Numb_2\PlosBiology\wjedrzej1@gmail.com)

**S6 Table. Estimates of jaguar populations within protected areas by current range countries.**

| Nr | Country | Protected Area (thousands km^2^) | Estimate of jaguar population (95% LCI - UCI) | Mean density  N/100 km^2^ (95% LCI - UCI) |
| --- | --- | --- | --- | --- |
| 1 | Brazil | 2,160.7 | 46,391 (35,702 - 56,361) | 2.15 (1.65 - 2.61) |
| 2 | Peru | 289.9 | 9,448 (7,484 - 11,542) | 3.26 (2.58 - 3.98) |
| 3 | Colombia | 219.3 | 4,492 (3,055 - 5,898) | 2.05 (1.39 - 2.69) |
| 4 | Bolivia | 179.4 | 3,490 (2,794 - 4,212) | 1.95 (1.56 - 2.35) |
| 5 | Venezuela | 322.6 | 6,769 (5,167 - 8,351) | 2.10 (1.60 - 2.59) |
| 6 | Guyana | 18.5 | 440 (324 - 552) | 2.38 (1.75 - 2.98) |
| 7 | Suriname | 23 | 535 (383 - 682) | 2.33 (1.67 - 2.97) |
| 8 | Ecuador | 23.3 | 523 (423 - 624) | 2.24 (1.82 - 2.68) |
| 9 | French Guiana | 43.7 | 852 (574 - 1123) | 1.95 (1.31 - 2.57) |
| 10 | Paraguay | 18 | 209 (109 - 308) | 1.16 (0.61 - 1.71) |
| 11 | Argentina | 19.5 | 141 (55 - 238) | 0.72 (0.28 - 1.22) |
| 13 | Uruguay | 0 | 0 (0 - 0) | 0 (0 - 0) |
| 12 | Chile | 0 | 0 (0 - 0) | 0 (0 - 0) |
|  | Total South America | 3,317.9 | 73,289 (56,070 - 89,891) | 2.21 (1.69 - 2.71) |
| 14 | Mexico | 56 | 1,060 (844 - 1,295) | 1.89 (1.51 - 2.31) |
| 15 | Nicaragua | 32.2 | 808 (650 - 979) | 2.51 (2.02 - 3.04) |
| 16 | Honduras | 25 | 737 (602 - 876) | 2.95 (2.41 - 3.5) |
| 17 | Guatemala | 25 | 701 (574 - 832) | 2.80 (2.30 - 3.33) |
| 18 | Panama | 16.8 | 346 (270 - 425) | 2.06 (1.61 - 2.53) |
| 19 | Costa Rica | 11.2 | 183 (142 - 235) | 1.63 (1.27 - 2.1) |
| 20 | Belize | 7.9 | 239 (196 - 284) | 3.03 (2.48 - 3.59) |
| 21 | United States | 0.5 | 0 (0 - 0) | 0 (0 - 0) |
| 22 | El Salvador | 0 | 0 (0 - 0) | 0 (0 - 0) |
|  | Total North America | 1,74.6 | 4,075 (3,278 - 4,926) | 2.33 (1.88 - 2.82) |
|  | Total Neotropics | 3,492.5 | 77,364 (62,090 - 92,951) | 2.22 (1.78 - 2.66) |
