# Supplementary material for: Estimating large carnivore populations at global scale based on spatial predictions of density and distribution – Application to the jaguar (Panthera onca)
Source: PLoS One. 2018 Mar 26;13(3):e0194719. doi: 10.1371/journal.pone.0194719 (PMC5868828; doi:10.1371/journal.pone.0194719)
Supplement: S1 Text — (DOCX) [file pone.0194719.s007.docx]

**Estimating large carnivore populations at global scale based on spatial predictions of density and distribution – application to the jaguar (*Panthera onca*)**

Jędrzejewski W.*, Robinson H.S., Abarca M., Zeller K.A., Velasquez G., Paemelaere E.A.D., Goldberg J.F., Payan E., Hoogesteijn R., Boede E.O., Schmidt K., Lampo M., Viloria Á.L., Carreño R., Robinson N., Lukacs P.M., Nowak J.J., Salom-Pérez R., Castañeda F., Boron V., Quigley H.

*correspondence to: [wjedrzej1@gmail.com](file:///C:\MDoc-Venezuela-S\Papers-manuscripts\Jaguar_Americas_Distr_Dens_Numb_2\PlosBiology\wjedrzej1@gmail.com)

**S1 Text. Discussion of possible effect of increased pixel size of covariate layers on the precision of population size estimates.**

It was not possible to run Markov chain Monte Carlo computations with 100,000 iterations and a pixel size 1 km x 1 km (originally used for our occurrence and density models). To make our analysis computationally feasible we reduced the number of pixels by resampling our covariate layers to 10 km x 10 km pixels. This change of cell size produces a slightly different population estimate depending on the heterogeneity and quality of local habitat [1,2]. For instance in the areas with prevailing favourable habitats the population estimate may be inflated, due to dissolution of smaller patches of poor habitat (e.g. human settlements). Conversely, in the areas where unfavourable habitat prevails (e.g. dry areas) increased pixel size could lower the population estimate due to dissolution of patches of good habitat (e.g. strips of gallery forests). To examine this possible change we ran a simplified analysis in which we multiplied density and occurrence models, using covariate layers with 1 km x 1 km pixel size. With this approach we obtained finer grained mean values, however without confidence intervals. The total estimated jaguar population size was only slightly different (4% lower) than that estimated at 100 km^2^ scale. However, for some countries, like Mexico or Colombia, this finer scale analysis produced estimates different by up to 13% (Table S1.1).

**Table S1.1.** **Mean estimates of population size, and mean density of jaguars in countries of South America and North America, based on multiplication of the density and occurrence models and 1 km x 1 km pixel size.**

| NP | Country | IUCN 2014 Jaguar Range Area (thousands km^2^) | Mean estimate of jaguar population size | Mean jaguar density N/100km^2^ |
| --- | --- | --- | --- | --- |
| 1 | Brazil | 4,541 | 82,373 | 1.81 |
| 2 | Peru | 741 | 22,973 | 3.10 |
| 3 | Colombia | 864 | 14,366 | 1.66 |
| 4 | Bolivia | 770 | 13,085 | 1.70 |
| 5 | Venezuela | 596 | 10,633 | 1.78 |
| 6 | Guyana | 211 | 3,948 | 1.87 |
| 7 | Suriname | 142 | 2,851 | 2.01 |
| 8 | Ecuador | 91 | 1,937 | 2.12 |
| 9 | Paraguay | 254 | 1,747 | 0.69 |
| 10 | French Guiana | 83 | 1,381 | 1.66 |
| 11 | Argentina | 80 | 308 | 0.38 |
|  | **Total South America** | **8,374** | **155,602** | **1.86** |
| 12 | Mexico | 379 | 4,683 | 1.24 |
| 13 | Nicaragua | 61 | 1,495 | 2.45 |
| 14 | Honduras | 50 | 1,215 | 2.44 |
| 15 | Guatemala | 45 | 1,082 | 2.42 |
| 16 | Panama | 44 | 948 | 2.17 |
| 17 | Belize | 22 | 616 | 2.83 |
| 18 | Costa Rica | 40 | 538 | 1.35 |
|  | **Total North America** | **640** | **10,577** | **1.65** |
|  | **Total Neotropics** | **9,014** | **166,179** | **1.84** |

**References for S1 Text:**

1. Wiens JA, Milne BT. Scaling of ‘landscapes’ in landscape ecology, or, landscape ecology from a beetle's perspective. Landsc Ecol. 1989; 3: 87-96.
2. Turner MG, O'Neill RV, Gardner RH, Milne BT. Effects of changing spatial scale on the analysis of landscape pattern. Landsc Ecol. 1989; 3: 153-162.
