# Supplementary material for: Estimating large carnivore populations at global scale based on spatial predictions of density and distribution – Application to the jaguar (Panthera onca)
Source: PLoS One. 2018 Mar 26;13(3):e0194719. doi: 10.1371/journal.pone.0194719 (PMC5868828; doi:10.1371/journal.pone.0194719)
Supplement: S2 Text — (DOC) [file pone.0194719.s008.doc]

**Estimating large carnivore populations at global scale based on spatial predictions of density and distribution – application to the jaguar (*Panthera onca*)**

Jędrzejewski W.*, Robinson H.S., Abarca M., Zeller K.A., Velasquez G., Paemelaere E.A.D., Goldberg J.F., Payan E., Hoogesteijn R., Boede E.O., Schmidt K., Lampo M., Viloria Á.L., Carreño R., Robinson N., Lukacs P.M., Nowak J.J., Salom-Pérez R., Castañeda F., Boron V., Quigley H.

*correspondence to: [wjedrzej1@gmail.com](../../../../C:%5CMDoc-Venezuela-S%5CPapers-manuscripts%5CJaguar_Americas_Distr_Dens_Numb_2%5CPlosBiology%5Cwjedrzej1@gmail.com)

S2 Text. Simulation to validate hierarchical model; methods and associated R code

We used simulation to validate our method of estimating the global population size of jaguars by creating a data set that resembled our real data set, but in which the true population size was known [1]. Our goal was to determine how well our method performed under similar data structure and sampling levels as the empirical data.

We performed our simulation in the following steps:

1)      To set the simulation to the size of our true study area we generated a matrix with 175,000 empty rows (equivalent to 175,000 grid cells, 100 km^2^ each, in the raster files we used for estimating the jaguar population).

2)      To each matrix row we assigned a column of random values representing covariates from our density and occurrence models (6 continuous covariates with values between -1 and 1, equivalent to the z transformed covariates that we used in our true estimation procedure, plus 1 dummy coded covariate matching our North America – South America and protected area binomial coded variables).

3)      To each row we randomly assigned values representing jaguar density and distribution using a random poison distribution where lambda equalled 1. This random population generation resulted in approximately half the cells being empty, and half having a density of between 1 and 8 “jaguars”, roughly representing the values observed from our true jaguar population (see Results in the main text).

4)      To simulate our true sampling effort we randomly selected 0.05% of the matrix rows for density (approximately 80 density samples) and 2% of the rows for occurrence (approximately 3,400 presence/absence points).

5) Finally we ran this simulated data through our hierarchical model using the same coding as with the empirical data (100,000 iterations, 1,000 burn in, 100 thinning, see Materials and Methods). We replicated this simulation procedure 100 times. In each simulation replicate, new “population” and covariate values were generated, sampled, and modelled. We used the outputs from these 100 replicates to test our method’s bias (mean error; ME), and accuracy (mean absolute error; MAE) [2].

R and JAGS code is provided below.

Our simulation method created an average “population” of 175,021 (SD = 439) animals across our 175,000 cells. Our mean estimated population resulting from our MCMC process was 171,560 (SE = 1405). All estimates produced by our hierarchical model were within +/-33,338 individuals of the true value (range 19 to 33,338, Fig S2.1). The model showed a slight negative bias where modelled estimates were lower than the true population (ME = -3,460) and good accuracy (MAE = 11,544). Our simulations suggest that this method and sampling level were sufficient to estimate the true jaguar population level with a reasonable degree of error.

**References for S2 Text:**

1. Kéry M, Royle JA. Applied Hierarchical Modeling in Ecology: Analysis of distribution, abundance and species richness in R and BUGS: Volume 1: Prelude and Static Models: Academic Press; 2016.
2. Walther BA, Moore JL. The concepts of bias, precision and accuracy, and their use in testing the performance of species richness estimators, with a literature review of estimator performance. Ecography. 2005; 28: 815-29.


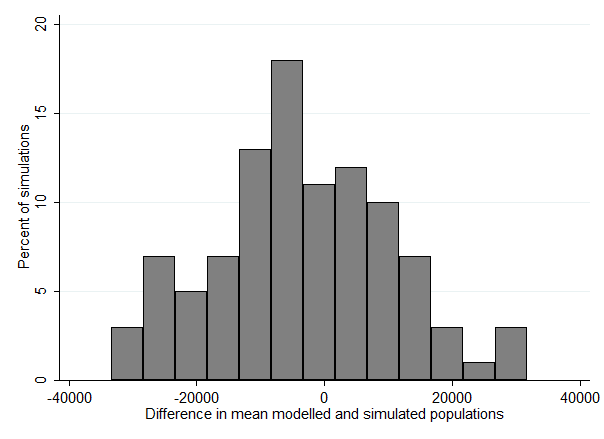


**Fig S2.1. Difference in mean modelled population estimate and true population for each of 100 simulation replicates (bin width = 5000).**

**R code used to: (1) create a simulated data set, (2) analyse the data using the same hierarchical model (and coding) used in our main analysis.**

##################################################################################

#SIMULATION OF OCCURRENCE AND DENSITY DATA FOR POPULATION ESTIMATION #

##################################################################################

library(dplyr) #used to subsample and manipulate data

library(R2jags) #to communicate with jags

library(mcmcplots) # to view diagnostic plots

#set the desired size or number of cells in the “study area”

cells <- 175000

#create spatial variables

#for continuous variables we use z transformed covs in Jags so here we use a

#normal distribution with mean 0 and standard deviation of 1.

#to match the structure of our real model we need 7 variables with 1 dummy coded

var1<-rnorm(n=cells, 0,1) # format is (number of obs, mean, SD)

var2<-rnorm(n=cells, 0,1)

var3<-rnorm(n=cells, 0,1)

var4<-rnorm(n=cells, 0,1)

var5<-rnorm(n=cells, 0,1)

var6<-rnorm(n=cells, 0,1)

var7<-rbinom(n=cells, 1 ,0.3) #format is (number of obs, attempts per ob, odds of success)

#create a population based on a random poison distribution

N<-(rpois(n=cells, lambda=1)) #poison distribution lambda is mean and variance

realpop<-sum(N) #total population size

table(N) #summary of the frequency distribution

hist(N) #just because I like histograms

#put the data together into a single data frame called truth

truth<-as.data.frame(cbind(N, var1, var2, var3, var4, var5, var6, var7))

truth<-mutate(truth, presence = ifelse(N<1,0,1)) #create a presence/absence column

truth<-mutate(truth, x=row_number()) #create a column of row ID numbered sequentially

#sample created population

denj <- 0.0005*cells # of cells sampled density estimates for the study area (i.e. how many cells did we survey)

occj <- 0.020*cells # of cells sampled for occupancy

#add columns that will represent our sampled values of density and occurrence

temp1<-mutate(truth, den_sample = N)

temp2<-mutate(temp1, occ_sample = ifelse(N==0,0,1))

#use sample_n to randomly sample a number of the temp2 values and fill sample columns with only #those values

temp3 <- sample_n(temp2, denj, replace=FALSE)

den_sample<-temp3[c("den_sample","x")]

temp4<-sample_n(temp2, occj, replace=FALSE)

occ_sample<-temp4[c("occ_sample","x")]

temp5<-left_join(truth, occ_sample, by="x")

simdata<-left_join(temp5, den_sample, by="x")

rm("temp1","temp2","temp3","temp4", "temp5"); gc()

#now pass simulated data (simdata) to our hierarchical/jags model to estimate total population

#############################################################################################

#HIERARCHICAL MODEL FOR POPULATION ESTIMATION BASED ON DENSITY AND DISTRIBUTION

#############################################################################################

den=simdata$den_sample

occ=simdata$occ_sample

#jags wants a list of variables

sp.data <- list(den=den, occ=occ, var1=simdata$var1, var2=simdata$var2, var3=simdata$var3,

var4=simdata$var4, var5=simdata$var5, var6=simdata$var6, var7=simdata$var7, nCells=nrow(simdata))

#Specify the parameters to be monitored

sp.params = c("a0","a1","a2", "a3", "a4",

"b0","b1","b2", "b3", "b4", "b5", "b6", "b7", "aveDen", "Ntot", "tau")

#Specify the initial values

sp.inits = function() {

list(a0 = runif(1,-1,1),

a1 = runif(1,-1,1),

a2 = runif(1,-1,1),

a3 = runif(1,-1,1),

a4 = runif(1,-1,1),

b0 = runif(1,-1,1),

b1 = runif(1,-1,1),

b2 = runif(1,-1,1),

b3 = runif(1,-1,1),

b4 = runif(1,-1,1),

b5 = runif(1,-1,1),

b6 = runif(1,-1,1),

b7 = runif(1,-1,1),

tau = c(101,0.1)

)

}

#Run the model and call the results

#jags text file must be in working directory

jag.res = jags(sp.data, sp.inits, sp.params, "jaguar_jags_validation.txt",

n.chains=1, n.iter=100000, n.burnin=1000, n.thin=100)

mcmcplot(jag.res)

Ntotal<-jag.res$BUGSoutput$sims.list$Ntot

summary(jag.res$BUGSoutput$sims.list$Ntot)

simsoutput<-as.data.frame(jag.res$BUGSoutput$sims.list)

write.csv(simsoutput, "D:/") #if you wish to keep the jags output

**#Jags code**

**#**in R working directory as “jaguar_jags_validation.txt”

model{

b0 ~ dnorm( 0, 0.001 ) # priors for occupancy model

b1 ~ dnorm( 0, 0.001 )

b2 ~ dnorm( 0, 0.001 )

b3 ~ dnorm( 0, 0.001 )

b4 ~ dnorm( 0, 0.001 )

b5 ~ dnorm( 0, 0.001 )

b6 ~ dnorm( 0, 0.001 )

b7 ~ dnorm( 0, 0.001 )

a0 ~ dnorm( 0, 0.001 ) # priors for abundance model

a1 ~ dnorm( 0, 0.001 )

a2 ~ dnorm( 0, 0.001 )

a3 ~ dnorm( 0, 0.001 )

a4 ~ dnorm( 0, 0.001 )

tau[1] ~ dunif( 100, 110 )

tau[2] ~ dunif( 0, 10 )

for( i in 1:nCells ){

# occurance model

logit(psi[i]) <- b0 + b1*var1[i] + b2*var2[i] + b3*var3[i] + b4*var4[i] + b5*var5[i] + b6*var6[i]+

b7*var7[i]

occ[i] ~ dbern( psi[i] )

tempOcc[i] <-occ[i]+1

# density model

mu[i,2] <- (a0 + a1*var1[i] + a2*var2[i]+ a3*var3[i] + a4*var7[i])

mu[i,1] <- 0

den[i] ~ dnorm( mu[i, tempOcc[i]], tau[tempOcc[i]] )

denReal[i] <- den[i]

}

aveDen <- sum(denReal)/(nCells)

Ntot <- aveDen*nCells

}
