# Supplementary material for: Estimating large carnivore populations at global scale based on spatial predictions of density and distribution – Application to the jaguar (Panthera onca)
Source: PLoS One. 2018 Mar 26;13(3):e0194719. doi: 10.1371/journal.pone.0194719 (PMC5868828; doi:10.1371/journal.pone.0194719)

**Estimating large carnivore populations at global scale based on spatial predictions of density and distribution – application to the jaguar (*Panthera onca*)**

Jędrzejewski W.*, Robinson H.S., Abarca M., Zeller K.A., Velasquez G., Paemelaere E.A.D., Goldberg J.F., Payan E., Hoogesteijn R., Boede E.O., Schmidt K., Lampo M., Viloria Á.L., Carreño R., Robinson N., Lukacs P.M., Nowak J.J., Salom-Pérez R., Castañeda F., Boron V., Quigley H.

*correspondence to: [wjedrzej1@gmail.com](file:///C:\MDoc-Venezuela-S\Papers-manuscripts\Jaguar_Americas_Distr_Dens_Numb_2\PlosBiology\wjedrzej1@gmail.com)

**S3 Fig. Spatial distribution of the posterior standard deviations of adjusted jaguar density estimates.**


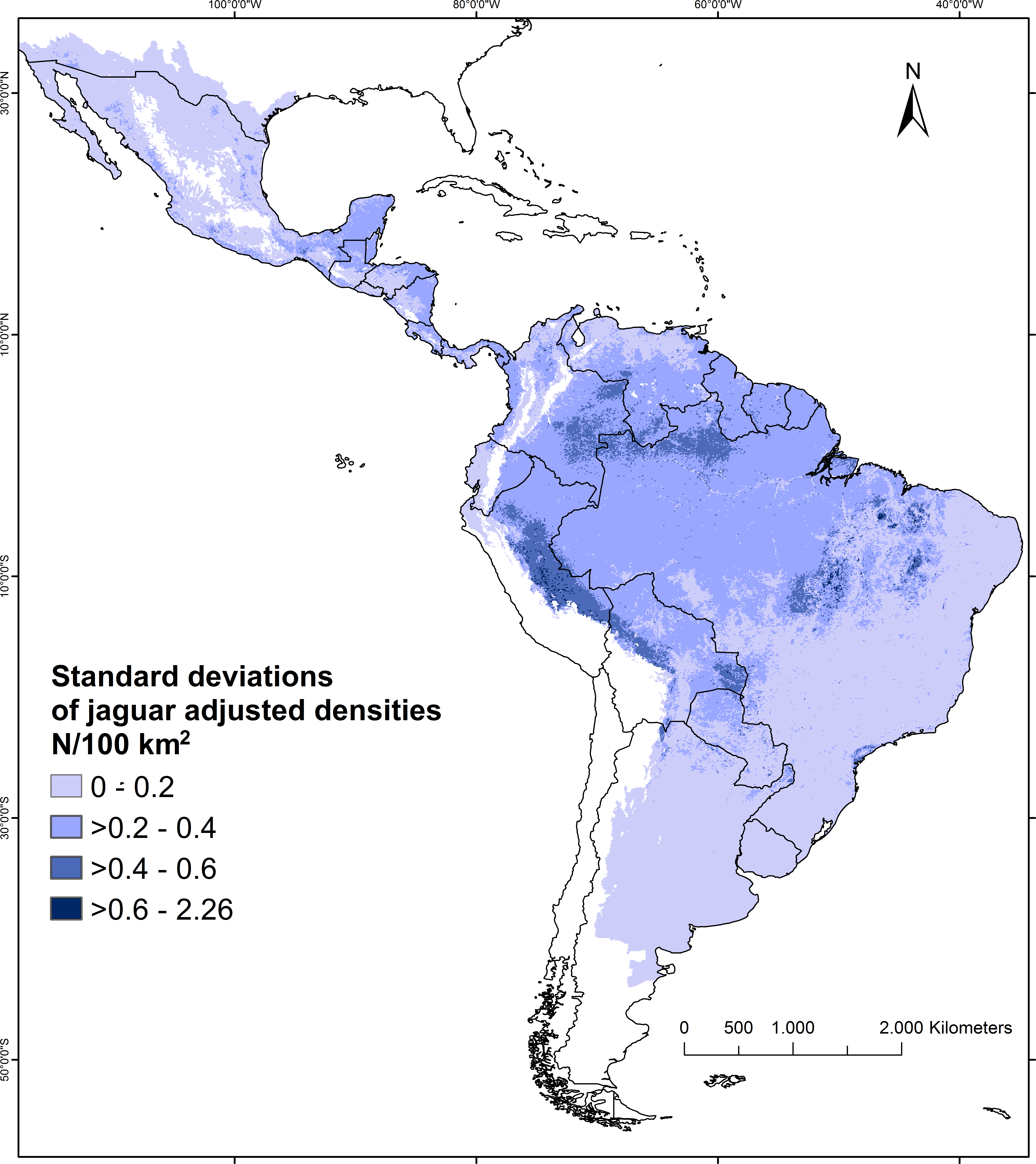

Supplement: S3 Fig — (DOCX) [file pone.0194719.s011.docx]
